# Smoothing of Binary Codes, Uniform Distributions, and Applications

**DOI:** 10.3390/e25111515

**Published:** 2023-11-05

**Authors:** Madhura Pathegama, Alexander Barg

**Affiliations:** Department of ECE and Institute for Systems Research, University of Maryland, College Park, MD 20742, USA; pankajap@umd.edu

**Keywords:** noise operator, uniform distribution, Renyi divergence, wiretap channel

## Abstract

The action of a noise operator on a code transforms it into a distribution on the respective space. Some common examples from information theory include Bernoulli noise acting on a code in the Hamming space and Gaussian noise acting on a lattice in the Euclidean space. We aim to characterize the cases when the output distribution is close to the uniform distribution on the space, as measured by the Rényi divergence of order α∈(1,∞]. A version of this question is known as the channel resolvability problem in information theory, and it has implications for security guarantees in wiretap channels, error correction, discrepancy, worst-to-average case complexity reductions, and many other problems. Our work quantifies the requirements for asymptotic uniformity (perfect smoothing) and identifies explicit code families that achieve it under the action of the Bernoulli and ball noise operators on the code. We derive expressions for the minimum rate of codes required to attain asymptotically perfect smoothing. In proving our results, we leverage recent results from harmonic analysis of functions on the Hamming space. Another result pertains to the use of code families in Wyner’s transmission scheme on the binary wiretap channel. We identify explicit families that guarantee strong secrecy when applied in this scheme, showing that nested Reed–Muller codes can transmit messages reliably and securely over a binary symmetric wiretap channel with a positive rate. Finally, we establish a connection between smoothing and error correction in the binary symmetric channel.

## 1. Introduction

Many problems of information theory involve the action of a noise operator on a code distribution, transforming it into some other distribution. For instance, one can think of Bernoulli noise acting on a code in the Hamming space or Gaussian noise acting on a lattice in the Euclidean space. We are interested in characterizing the cases when the output distribution is close to the uniform distribution on the space. Versions of this problem have been considered under different names, including resolvability [1,2,3], smoothing [4,5], discrepancy [6,7], and the entropy of noisy functions [8,9,10]. Direct applications of smoothing include secrecy guarantees in both the binary symmetric wiretap channel [2,3,11] and the Gaussian wiretap channel [12,13], error correction in the binary symmetric channel (BSC) [14,15], converse coding theorems of information theory [1,16,17,18], strong coordination [11,19,20,21,22], secret key generation [13,23], and worst-to-average case reductions in cryptography [5,24]. Some aspects of this problem also touch upon approximation problems in statistics and machine learning [25,26,27].

Our main results are formulated for the smoothing in the binary Hamming space $H_{n}$. For $r:H_{n}\to R_{0}^{+}$, and $f:H_{n}\to R$ define

$$T_{r}f\left(x\right)=\left(r*f\right)\left(x\right):=\sum_{z\in H_{n}}r\left(z\right)f\left(x-z\right)$$

as the action of *r* on the functions on the space. We set *r* to be a probability mass function (pmf) and call the function $T_{r}f$ the *noisy version* of *f* with respect to *r*, and refer to *r* and $T_{r}$ as a *noise kernel* and a *noise operator*, respectively. By *smoothing f* with respect to *r*, we mean applying the noise kernel *r* to *f*. We often assume that r(x) is a radial kernel, i.e., its value on the argument $x\in H_{n}$ depends only on the Hamming weight of *x*.

There are several ways to view the smoothing operation. Interpreting it as a shift-invariant linear operator, we note that, from Young’s inequality, $∥T_{r}{f∥}_{\alpha }={∥f*r∥}_{\alpha }\leq {∥f∥}_{\alpha },1\leq \alpha \leq \infty ,$ so smoothing contracts the α-norm. Upon applying $T_{r}$, the noisy version of *f* becomes “flatter”; hence, the designation “smoothing”. Note that if *f* is a pmf, then $T_{r}f$ is also a pmf, and so this view allows us to model the effect of communication channels with additive noise.

The class of functions that we consider are (normalized) indicators of subsets (codes) in $H_{n}$. A code $C\subset H_{n}$ defines a pmf $f_{C}=\frac{1_{C}}{\left|C\right|},$ and, thus, $T_{r}f_{C}$ can be viewed as a noisy version of the code (we also sometimes call it a *noisy distribution*) with respect to the kernel *r*. The main question of interest for us is the proximity of this distribution to $U_{n},$ or the “smoothness” of the noisy code distributions. To quantify closeness to $U_{n}$, we use the Kullback–Leibler (KL) and Rényi divergences (equivalently, $L_{\alpha }$ norms), and the smoothness measured in $D_{\alpha }\left(\cdot ∥\cdot \right)$ is termed the $D_{\alpha }$-smoothness ($L_{\alpha }$-smoothness).

We say that a code is *perfectly smoothable* with respect to the noise kernel *r* if the resultant noisy distribution becomes uniform. Our main emphasis is on the asymptotic version of perfect smoothing and its implications for some of the basic information-theoretic problems. A sequence of codes ${\left(C_{n}\right)}_{n}$ is asymptotically smoothed by the kernel sequence $r_{n}$ if the distance between $\left(T_{r_{n}}f_{C_{n}}\right)$ and $U_{n}$ approaches 0 as *n* increases. This property is closely related to the more general problem of *channel resolvability* introduced by Han and Verdú in [1]. Given a discrete memoryless channel W(Y|X) and a distribution $P_{X}$, we observe a distribution $P_{Y}$ on the output of the channel. The task of channel resolvability is to find $P_{X}$ supported on a subset $C\subset H_{n}$ that approximates $P_{Y}$ with respect to the KL divergence. As shown in [1], there exists a threshold value of the rate such that it is impossible to approximate $P_{Y}$ using codes of lower rate, while any output process can be approximated by a well-chosen code of a rate larger than the threshold. Other proximity measures between distributions were considered for this problem in [3,28,29]. Following the setting in [3], we consider Rényi divergences for measuring the closeness to uniformity. We call the minimum rate required to achieve perfect asymptotic smoothing the *$D_{\alpha }$-smoothing capacity* of the noise kernels ${\left(r_{n}\right)}_{n}$, where the proximity to uniformity is measured by the α-Rényi divergence. In this work, we characterize the $D_{\alpha }$-smoothing capacity of the sequence ${\left(r_{n}\right)}_{n}$ using its Rényi entropy rate.

*Asymptotic smoothing.* We will limit ourselves to studying smoothing bounds under the action of the Bernoulli noise or ball noise kernels, defined formally below. A common approach to deriving bounds on the norm of a noisy function is through hypercontractivity inequalities [30,31,32]. In its basic version, given a code C of size *M*, it yields the estimate

$$\begin{array}{c} ∥T_{\delta }f_{C}{∥}_{\alpha }\leq {∥f_{C}∥}_{\alpha^{\prime }}=M^{\frac{1-\alpha^{\prime }}{\alpha^{\prime }}}2^{-\frac{n}{\alpha^{\prime }}}, \end{array}$$

where $T_{\delta }$ is the Bernoulli kernel (see Section 2 for formal definitions) and $\alpha^{\prime }=1+{\left(1-2\delta \right)}^{2}\left(\alpha -1\right)$. This upper bound does not differentiate codes yielding higher or lower smoothness, which in many situations may not be sufficiently informative. Note that other tools, such as “Mrs. Gerber’s lemma” [30,33] or strong data-processing inequalities, also suffer from the same limitation.

A new perspective of the bounds for smoothing has recently been introduced in the works of Samorodnitsky [8,9,10]. Essentially, his results imply that codes satisfying certain regularity conditions have good smoothing properties. Their efficiency is highlighted in recent papers [14,34], which leveraged results for code performance on the binary erasure channel (BEC) to prove strong claims about the error correction capabilities of the codes when used on the BSC. Using Samorodnitsky’s inequalities, we show that the duals of some BEC capacity-achieving codes achieve $D_{\alpha }$-smoothing capacity for α∈{2,3,…,∞} with respect to the Bernoulli noise. This includes the duals of polar codes and doubly transitive codes, such as the Reed–Muller (RM) codes.

*Smoothing and the wiretap channel.* Wyner’s wiretap channel [35] models communication in the presence of an eavesdropper. Code design for this channel pursues reliable communication between the legitimate parties, while at the same time leaking as little information as possible about the transmitted messages to the eavesdropper. The connection between secrecy in wiretap channels and resolvability was first mentioned by Csiszár [36] and later developed by Hayashi [2]. It rests on the observation that to achieve secrecy it suffices to make the distribution of an eavesdropper’s observations conditioned on the transmitted message nearly independent of the message. The idea of characterizing secrecy based on smoothness works irrespective of the measure of secrecy [2,3,11], and it was also employed for nested lattice codes used over the Gaussian wiretap channel in [12].

Secrecy on the wiretap channel can be defined in two ways, measured by the information gained by the eavesdropper, and it depends on whether this quantity is normalized to the number of channel uses (weak secrecy) or not (strong secrecy). This distinction was first highlighted by Maurer [37], and it has been adopted widely in the recent literature. Early papers devoted to code design for the wiretap channel relied on random codes, but, for simple channel models such as BSC or BEC, this has changed with the advent of explicit capacity-approaching code families. Weak secrecy results based on LDPC codes were presented in [38], but initial attempts to attain strong secrecy encountered some obstacles. To circumvent this, the first works on code construction [39,40] had to assume that the main channel is noiseless. The problem of combining strong secrecy and reliability for general wiretap channels was resolved in [41], but that work had to assume that the two communicating parties share a small number of random bits unavailable to the eavesdropper. Apart from the polar coding scheme of [41], explicit code families that support reliable communication with positive rate and strong secrecy have not previously appeared in the literature. In this work, we show that nested RM codes perform well in binary symmetric wiretap channels based on their smoothing properties. While our work falls short of proving that nested RM codes achieve capacity, we show that they can transmit messages reliably and secretly at rates close to capacity.

*Ball noise and decoding error.* Ball-noise smoothing provides a tool for estimating the error probability of decoding on the BSC. We derive impossibility and achievability bounds for the $D_{\alpha }$-smoothness of noisy distributions with respect to the ball noise. Smoothing of a code with respect to the $L_{2}$ norm plays a special role because, in this case, the second norm (the variance) of the resulting distribution can be expressed via the pairwise distance between codewords, enabling one to rely on tools from Fourier analysis. The recent paper by Debris-Alazard et al. [4] established universal bounds for the smoothing of codes or lattices, with cryptographic reductions in mind. The paper by Sprumont and Rao [15] addressed bounds for error probability of list decoding at rates above BSC capacity. A paper by one of the present authors [42] studied the variance of the number of codewords in balls of different radii (a quantity known as the quadratic discrepancy [43,44]).

The main contributions of this paper are the following:

1. Characterizing the $D_{\alpha }$-smoothing capacities of noise operators on the Hamming space for α∈(1,∞].
2. Identifying some explicit code families that attain a smoothing capacity of the Bernoulli noise for α∈{2,3,…,∞};
3. Obtaining rate estimates for the RM codes used on the BSC wiretap channel under the strong secrecy condition;
4. Showing that codes possessing sufficiently good smoothing properties are suitable for error correction.

In Section 2, we set up the notation and introduce the relevant basic concepts. Then, in Section 3, we derive expressions for the $D_{\alpha }$-smoothing capacities for α∈(1,∞], and in Section 4, we use these results to analyze the smoothing of code families under the action of the Bernoulli noise. Section 5 is devoted to the application of these results for the binary symmetric wiretap channel. In particular, we show that RM codes can achieve rates close to the capacity of the BSC wiretap channel, while at the same time guaranteeing strong secrecy. In Section 6, we establish threshold rates for smoothing under ball noise, and derive bounds for the error probability of decoding on the BSC, including the list case, based on the distance distribution. Concluding the paper, Section 7 briefly points out that the well-known class of uniformly packed codes are perfectly smoothable with respect to “small” noise kernels.

## 2. Preliminaries

### 2.1. Notation

Throughout this paper, $H_{n}$ is the binary *n*-dimensional Hamming space

*Balls and spheres.* Denote by $B\left(x,t\right):=\{y\in H_{n}:|y-x|\leq t\}$ the metric ball of radius *t* in $H_{n}$ with center at *x*, and denote by $S\left(x,t\right):=\{y\in H_{n}:|y-x|=t\}$ the sphere of radius *t*. Let $V_{t}=\left|B\left(x,t\right)\right|$ be the volume of the ball, and let $\mu_{t}\left(i\right)$ be the intersection volume of two balls of radius *t* whose centers are distance *i* apart:

(1) $$\begin{array}{c} \mu_{t}\left(i\right)=\left|B\left(0,t\right)\cap B\left(x,t\right)|,\;\mathrm{where}\;|x\right|=i. \end{array}$$

*Codes and distributions.* A code C is a subset in $H_{n}$. The rate and distance of the code are denoted by R(C):=log|C|/n and d(C), respectively. Let

(2) $$A_{i}=\frac{1}{\left|C\right|}\left|\left\{\left(x,y\right)\in C^{2}:d\left(x,y\right)=i\right\}\right|$$

and let $\left(A_{i},i=0,\ldots ,n\right)$ be the distance distribution of the code. If the code C forms an $F_{2}$-linear subspace in $H_{n},$ we denote by $C^{⊥}:=\left\{y\in H_{n}:\sum_{i}x_{i}y_{i}=0\;\mathrm{for}\;\mathrm{all}\;x\in C\right\}$ its dual code.

The function $1_{C}$ denotes the indicator of a subset $C\subset H_{n},$ and $f_{C}=\frac{1_{C}}{\left|C\right|}$ is the corresponding pmf denoting the uniform distribution over the set, calling it a *code distribution*. Let $b_{t}$ denote the uniform distribution on the ball $B_{0,t}$, given by $b_{t}\left(x\right)=\frac{1_{B_{0},t}}{V_{t}}$. In the context of noise operators, we refer to $T_{b_{t}}$ as the *ball noise*. Finally, $\beta_{\delta }$ is the binomial distribution on $H_{n},$ given by

(3) $$\beta_{\delta }\left(x\right)=\beta_{\delta }^{\left(n\right)}\left(x\right)=\delta^{\left|x\right|}{\left(1-\delta \right)}^{n-|x|},$$

and $U_{n}$ is the uniform distribution, given by $U_{n}\left(x\right)=2^{-n}$ for all x.

*Entropies and norms.* For a function $f:H_{n}\to R$, we define its α-norm as follows.

$$\begin{array}{cc} {∥f∥}_{\alpha } & ={\left(\frac{1}{2^{n}}\sum_{x\in H_{n}}{\left|f(x)\right|}^{\alpha }\right)}^{1/\alpha }\;\mathrm{for}\;\alpha \in \left(0,\infty \right)\\ {∥f∥}_{\infty } & =\max_{x\in H_{n}}\left|f(x)\right|. \end{array}$$

Given a pmf *P*, let

(4) $$\begin{array}{cc} H(P) & =-\sum_{i}P_{i}\log P_{i}, \end{array}$$

(5) $$\begin{array}{cc} H_{\alpha }\left(P\right) & =\frac{1}{1-\alpha }\log\left(\sum_{i}P_{i}^{\alpha }\right) \end{array}$$

denote its Shannon entropy and Rényi entropy of order α, respectively. If *P* is supported on two points, we write h(P) and $h_{\alpha }\left(P\right)$ instead (all logarithms are to the base 2). The limiting cases of α=0,1,∞ are well-defined; in particular, for α=1, $H_{\alpha }\left(P\right)$ reduces to H(P).

For two discrete probability distributions *P* and *Q*, the α-*Rényi divergence* (or simply the α-divergence) is defined as follows:

(6) $$D_{\alpha }\left(P∥Q\right)=\left\{\begin{array}{cc} -\log Q(\left\{i:P_{i}>0\right\}) & \mathrm{if}\;\alpha =0\\ \frac{1}{\alpha -1}\log\sum_{i}P_{i}^{\alpha }Q_{i}^{-(\alpha -1)} & \mathrm{if}\;\alpha \in (0,1)\cup (1,\infty )\\ \sum_{i}P_{i}\log\frac{P_{i}}{Q_{i}} & \mathrm{if}\;\alpha =1\\ \max_{i}\log\frac{P_{i}}{Q_{i}} & \mathrm{if}\;\alpha =\infty \end{array}\right..$$

The divergence $D_{\alpha }\left(P∥Q\right)$ is a continuous function of α for α∈[0,∞]. For a pmf *f* on $H_{n}$

(7) $$\begin{array}{cc} D_{\alpha }\left(f∥U_{n}\right) & =\frac{\alpha }{\alpha -1}\log{∥2^{n}f∥}_{\alpha },\;\alpha \in \left(0,1\right)\cap \left(1,\infty \right) \end{array}$$

(8) $$\begin{array}{cc} D_{\infty }\left(f∥U_{n}\right) & =\log∥2^{n}{f∥}_{\infty }. \end{array}$$

Note that $D_{\alpha }\left(f∥U_{n}\right)=n-H_{\alpha }\left(f\right)$ for all 0≤α≤∞.

*Channels.* In this paper, a channel is a conditional probability distribution W:{0,1}→Y, where Y is a finite set, so that W(y|x) is the conditional probability of the output *y* for the input *x*. We frequently consider the binary symmetric channel with crossover probability δ and the binary erasure channel with erasure probability λ, abbreviating them as BSC(δ) and BEC(λ), respectively. We are often interested in the *n*-fold channel $W^{\left(n\right)},$ i.e., the conditional probability distribution corresponding to *n*-uses of the channel. For the input *X*, let $Y_{\left(X,W\right)}$ be the random output of the channel $W^{\left(n\right)}$. If the input sequences are chosen from a uniform distribution on a code C, we denote the input by $X_{C}.$ Since the number of uses of the channel is usually clear from the context, we suppress the dependency on *n* from the notation for channels and sequences.

Let C be a code of length *n*. For a channel W and input $X_{C}$, the block-MAP decoder is defined as

$$\begin{array}{c} \hat{x}\left(y\right)=\underset{x\in C}{\mathrm{error}}\mathrm{Pr}\left(x|y\right). \end{array}$$

For a given code and channel, denote the error probability of the block-MAP decoding by

$$\begin{array}{c} P_{B}\left(W,C\right)=\mathrm{Pr}(X_{C}\neq \hat{X}\left(Y_{\left(X_{C},W\right)}\right). \end{array}$$

### 2.2. $D_{\alpha }$- and $L_{\alpha }$-Smoothness

Recall that in the introduction, we expressed the smoothness of a distribution as its proximity to uniformity. Here, we formalize this notion based on two (equivalent) proximity measures.

Let *g* be a pmf on $H_{n}$. A natural measure of the uniformity of *g* is $D_{\alpha }\left(g∥U_{n}\right)$ (α∈[0,∞]). We call this the $D_{\alpha }$-smoothness of *g*. Observe that

(9) $$\begin{array}{cc} ∥2^{n}{g∥}_{\alpha } & =\frac{{∥g∥}_{\alpha }}{{∥g∥}_{1}}\geq 1\;\;\mathrm{for}\;\alpha \in \left(1,\infty \right],\;\mathrm{and}\; \end{array}$$

(10) $$\begin{array}{cc} ∥2^{n}{g∥}_{\alpha } & =\frac{{∥g∥}_{\alpha }}{{∥g∥}_{1}}\leq 1\;\;\mathrm{for}\;\alpha \in \left(0,1\right) \end{array}$$

with equality iff $g=U_{n}$. Thus, the better the pmf *g* approximates uniformity, the closer is $∥2^{n}{g∥}_{\alpha }$ to 1 (the denominator is simply a normalization quantity that allows dimension-agnostic analysis). Therefore, $∥2^{n}{g∥}_{\alpha }$ (α∈(0,1)∪(1,∞]) can be considered as another measure of proximity. We call $∥2^{n}{g∥}_{\alpha }$ the $L_{\alpha }$-smoothness of *g*. From (7) and (8), it follows that the $D_{\alpha }$-smoothness and $L_{\alpha }$-smoothness are equivalent.

**Remark 1.**  *It is easily seen that $D_{\alpha }\left(g∥U_{n}\right)=n-H_{\alpha }\left(g\right)$; hence, $D_{\alpha }\left(g∥U_{n}\right)$ is an increasing function of α.*

Recall that for a given code C, and a noise kernel *r*, $T_{r}f_{C}=r*f_{C}$ is the noisy distribution of code C with respect to *r*. We intend to study the smoothing properties of such noisy distributions of codes. In particular, we characterize the necessary conditions for $D_{\alpha }\left(T_{r}f_{C}∥U_{n}\right)$ to be close to zero (equivalently, for $∥2^{n}T_{r}f_{C}{∥}_{\alpha }$ close to one). In Section 3, we quantify these requirements in the asymptotic setting.

### 2.3. Resolvability

The problem of channel resolvability was introduced by Han and Verdú [1] under the name of approximating the output statistics of the channel. The objective of channel resolvability is to approximate the output distribution of a given input by the output distribution of a code with a smaller support size. In this work, we are interested in code families whose noisy distributions approximate uniformity. Resolvability characterizes the necessary conditions for this to happen in terms of the rate of the code.

Let W be a (discrete memoryless) channel whose input alphabet is X and whose output alphabet is Y. Let $X={\left\{X_{n}\right\}}_{n=1}^{\infty }$ be a discrete-time random process where the RVs $X_{n}$ take values in X. Denote by $Y_{n}$ the random output of W with input $X_{n}$ and let $Y={\left\{Y_{n}\right\}}_{n=1}^{\infty }.$ Denote by $P_{Y}$ the distribution of Y and let $P_{Y^{\left(n\right)}}$ be the pmf of the *n*-tuple $Y^{\left(n\right)}:=\left\{Y_{1},Y_{2},\ldots ,Y_{n}\right\}$.

For a legitimate (realizable) output process Y, define

(11) $$\begin{array}{c} J^{\left(\Delta \right)}\left(W,P_{Y}\right)=\underset{C_{n}\subset X^{n}}{\inf}\left\{\underset{n}{lim inf}R\left(C_{n}\right):\Delta \left(f_{C_{n}},P_{Y^{\left(n\right)}}\right)\to 0\right\}, \end{array}$$

where Δ is a measure of closeness of a pair of probability distributions. In words, we look for sequences of distributions ${\left(f_{C_{n}}\right)}_{n}$ of the smallest possible rate that approximates $P_{Y}$ on the output of W.

The original problem as formulated by Han and Verdú in [1] seeks to find the *resolvability* of the channel, defined as

(12) $$\begin{array}{c} C_{r}^{\left(\Delta \right)}\left(W\right)=\underset{P_{Y}}{\inf}\left\{J^{\left(\Delta \right)}\left(W,P_{Y}\right):Y\;\mathrm{is}\;\mathrm{an}\;\mathrm{output}\;\mathrm{process}\;\mathrm{over}\;W\right\}. \end{array}$$

where Δ is either the variational distance or the normalized KL divergence $\frac{1}{n}D\left(\cdot ∥\cdot \right).$ Hayashi [2] considered the same problem where the proximity was measured by the unnormalized KL divergence. In each case, the resolvability equals the Shannon capacity of the channel W.

**Theorem 1**  ([1,2]). *Let W be a discrete memoryless channel. Suppose that Δ is either the KL divergence (normalized or not) or the variational distance; then, the resolvability is given by*

$$\begin{array}{c} C_{r}^{\left(\Delta \right)}\left(W\right)=C\left(W\right), \end{array}$$

*where C(W) is the Shannon capacity of the channel.*

The authors of [1] proved this result under the additional assumption that the channel W satisfies strong converse, and Hayashi [2] later showed that this assumption is unessential.

In addition to the proximity measures considered in Theorem 1, the papers [3,28,29] considered other possibilities. In particular, Yu and Tan [3] studied the resolvability problem for a specific target distribution $P_{Y}$ and for the Rényi divergence $\Delta =D_{\alpha }$ (6). Their main result is as follows.

**Theorem 2**  ([3], Theorem 2). *Let W be a channel and $P_{Y}$ be an output distribution. then*

$$\begin{array}{c} J^{\left(D_{\alpha }\right)}\left(W,P_{Y}\right)=\left\{\begin{array}{cc} \min_{P_{X}\in P\left(W,P_{Y}\right)}\sum_{x}P_{X}\left(x\right)D_{\alpha }\left(W\left(\cdot |x\right)∥P_{Y}\right) & if\alpha \in (1,2]\cup \{\infty \}\\ \min_{P_{X}\in P\left(W,P_{Y}\right)}D(W∥P_{Y}\left|P_{X}\right) & if\alpha \in (0,1]\\ 0 & if\alpha =0, \end{array}\right. \end{array}$$

*where $P(W,P_{Y})$ is the set of distributions $P_{X}$ consistent with the output $P_{Y}$.*

A direct corollary of Theorem 2 is the following:

**Corollary 1**  ([3], Equation (55)). *Let $Y^{*}$ be the output process where for each n, $Y_{n}^{*}∼\mathrm{Ber}\left(1/2\right)$. Then,*

$$\begin{array}{c} J^{\left(D_{\alpha }\right)}\left(\mathrm{BSC}\left(\delta \right),P_{Y^{*}}\right)=\left\{\begin{array}{cc} 1-h_{\alpha }\left(\delta \right) & if\alpha \in (1,2]\cup \{\infty \}\\ 1-h(\delta ) & if\alpha \in (0,1]\\ 0 & if\alpha =0. \end{array}\right. \end{array}$$

This corollary gives necessary conditions for the rate of codes that can approximate the uniform distribution via smoothing. We will connect this result to the problem of finding smoothing thresholds in Section 4.

## 3. Perfect Smoothing—The Asymptotic Case

For a given family of noise kernels ${\left(T_{r_{n}}\right)}_{n}$, there exists a threshold rate such that it is impossible to approximate uniformity with codes of rate below the threshold irrespective of the chosen code, while at the same time, there exist families of codes with a rate above the threshold that allows perfect approximation in the limit of infinite length. For instance, for the Bernoulli(δ) noise applied to a code C, the smoothed distribution is nonuniform unless $C=H_{n}$ or δ=1/2. At the same time, it is possible to approach the uniform distribution asymptotically for large *n* once the code sequence satisfies certain conditions. Intuitively, it is clear that, for a fixed noise kernel, it is easier to approximate uniformity if the code rate is sufficiently high. In this section, we characterize the threshold rate for (asymptotically) perfect smoothing. Of course, the threshold also depends on the proximity measure Δ that we are using. In this section, we use perfect smoothing to mean “asymptotically perfect”. If the proximity measure Δ for smoothing is not specified, this means that we are using the KL divergence. We obtain the threshold rates for perfect smoothing measured with respect to the α-divergence for several values of α. In the subsequent sections, we work out the details for the Bernoulli and ball noise operators, which also have some implications for communication problems.

**Definition 1.**  *Let ${\left(C_{n}\right)}_{n}$ be a sequence of codes of increasing length n and let 0≤α≤∞. We say that the sequence $C_{n}$ is asymptotically perfectly $D_{\alpha }$-smoothable with respect to the noise kernels $r_{n}$ if*

$$\lim_{n\to \infty }D_{\alpha }\left(T_{r_{n}}f_{C_{n}}∥U_{n}\right)=0,$$

*or equivalently (7) and (8) if*

$$\lim_{n\to \infty }{∥2^{n}T_{r_{n}}f_{C_{n}}∥}_{\alpha }=1\;\left(\alpha \neq 0,1\right).$$

One can also define a dimensionless measure for perfect asymptotic smoothing by considering the limiting process

(13) $$\frac{∥T_{r_{n}}f_{C_{n}}-U_{n}{∥}_{\alpha }}{∥T_{r_{n}}f_{C_{n}}{∥}_{1}}=2^{n}{∥T_{r_{n}}f_{C_{n}}-U_{n}∥}_{\alpha }\to 0.$$

**Proposition 1.**  *Convergence in (13) implies perfect smoothing for all 1<α≤∞ and is equivalent to it for α≠∞.*

**Proof.**  Let $C=C_{n}\subset H_{n}$ for some fixed *n*. Since by the triangle inequality,

$$2^{n}∥T_{r}f_{C}{∥}_{\alpha }-1\leq 2^{n}{∥T_{r}f_{C}-U_{n}∥}_{\alpha },$$

(13) is not weaker than the mode of convergence in Definition 1 for all α∈[1,∞]. For α≠1,∞, we use Clarkson’s inequalities ([45], p. 388). Their form depends on α; namely, for 2≤α<∞, we have

$$\begin{array}{cc} 1+{∥\frac{2^{n}T_{r}f_{C}-1}{2}∥}_{\alpha }^{\alpha } & \leq {∥\frac{2^{n}T_{r}f_{C}+1}{2}∥}_{\alpha }^{\alpha }+{∥\frac{2^{n}T_{r}f_{C}-1}{2}∥}_{\alpha }^{\alpha }\\ \; & \leq \frac{1}{2}(∥2^{n}T_{r}f_{C}{∥}_{\alpha }^{\alpha }+1). \end{array}$$

For 1<α<2, the inequality has the form

$$\begin{array}{cc} 1+{∥\frac{2^{n}T_{r}f_{C}-1}{2}∥}_{\alpha }^{\alpha^{\prime }} & \leq {∥\frac{2^{n}T_{r}f_{C}+1}{2}∥}_{\alpha }^{\alpha^{\prime }}+{∥\frac{2^{n}T_{r}f_{C}-1}{2}∥}_{\alpha }^{\alpha^{\prime }}\\ \; & \leq {\left(\frac{1}{2}(∥2^{n}T_{r}f_{C}{∥}_{\alpha }^{\alpha }+1)\right)}^{\alpha^{\prime }/\alpha }, \end{array}$$

where $\alpha^{\prime }=\frac{\alpha }{\alpha -1}$ is the Hölder conjugate. These equations show that, for α∈(1,∞), $∥2^{n}T_{r_{n}}f_{C_{n}}{∥}_{\alpha }\to 1$ implies $∥2^{n}T_{r_{n}}f_{C_{n}}{-1∥}_{\alpha }\to 0,$ establishing the claimed equivalence. □

**Definition 2.**  *Let ${\left(r_{n}\right)}_{n}$ be a sequence of noise kernels. We say that the rate R is achievable for perfect $D_{\alpha }$-smoothing if there exists a sequence of codes ${\left(C_{n}\right)}_{n}$ such that $R(C_{n})\to R$ as n→∞ and ${\left(C_{n}\right)}_{n}$ is perfectly $D_{\alpha }$-smoothable.*

Note that if $R_{1}$ is achievable, then any rate $1\geq R_{2}>R_{1}$ is also achievable. Indeed, consider a (linear) code $C_{1}$ of rate $R_{1}$ that has good smoothing properties. Construct $C_{2}$ by taking the union of $2^{n(R_{2}-R_{1})}$ non-overlapping shifts of $C_{1}$. Then the rate of $C_{2}$ is $R_{2}$, and since each shift has good smoothing properties, the same is true for $C_{2}$. Therefore, let us define the main concept of this section.

**Definition 3.**  *Given a sequence of kernels $r={\left(r_{n}\right)}_{n}$, define the $D_{\alpha }$-smoothing capacity as*

(14) $$S_{\alpha }^{r}:=\underset{{\left(C_{n}\right)}_{n}}{\inf}\left\{\underset{n\to \infty }{lim inf}R\left(C_{n}\right):\lim_{n\to \infty }D_{\alpha }\left(T_{r_{n}}f_{C_{n}}∥U_{n}\right)=0\right\}.$$

Note that this quantity is closely related to the resolvability: if, rather than optimizing on the output process in (12), we set the output distribution to uniform and take $\Delta =D_{\alpha },$ then $S_{\alpha }^{r}$ equals $J^{\left(D_{\alpha }\right)}\left(W,P_{Y}\right)$ for the channel W given by the noise kernel r. To avoid future confusion, we refer to the capacity of reliable transmission as Shannon’s capacity.

The following lemma provides a lower bound for $D_{\alpha }$-smoothness. It follows from Lemma 2 in [3], and we give a direct proof for completeness.

**Lemma 1.**  *Let $C\subset H_{n}$ be a code of size $M=2^{nR}$ and let r be a noise kernel. Then, for α∈[0,∞]*

$$D_{\alpha }\left(T_{r}f_{C}∥U_{n}\right)\geq n\left(1-R\right)-H_{\alpha }\left(r\right).$$

**Proof.**  We will first prove that $∥2^{n}T_{r}f_{C}{∥}_{\alpha }^{\alpha }\geq 2^{\left(\alpha -1\right)[n\left(1-R\right)-H_{\alpha }\left(r\right)]}$ for α∈(1,∞):

$$\begin{array}{cc} ∥2^{n}T_{r}f_{C}{∥}_{\alpha }^{\alpha } & =\frac{2^{n\alpha }}{2^{n}}\sum_{x\in H_{n}}T_{r}f_{C}{\left(x\right)}^{\alpha }\\ \; & =2^{n(\alpha -1)}\sum_{x\in H_{n}}{\left[\sum_{y\in H_{n}}r\left(y\right)f_{C}\left(x-y\right)\right]}^{\alpha }\\ \; & \geq 2^{n(\alpha -1)}\sum_{x\in H_{n}}\sum_{y\in H_{n}}{\left[r\left(y\right)f_{C}\left(x-y\right)\right]}^{\alpha }\\ \; & =2^{n(\alpha -1)}\sum_{y\in H_{n}}r{\left(y\right)}^{\alpha }\sum_{x\in H_{n}}f_{C}{\left(x-y\right)}^{\alpha }\\ \; & =\frac{2^{n\alpha }}{{\left|C\right|}^{\left(\alpha -1\right)}}{∥r∥}_{\alpha }^{\alpha }\\ \; & =2^{\left(\alpha -1\right)[n\left(1-R\right)-H_{\alpha }\left(r\right)]}. \end{array}$$

Together with (7), this implies that the claimed inequality holds for α∈(1,∞).

A similar calculation shows that for α∈(0,1), $∥2^{n}T_{r}f_{C}{∥}_{\alpha }^{\alpha }\leq 2^{\left(\alpha -1\right)[n\left(1-R\right)-H_{\alpha }\left(r\right)]},$ yielding the claim for α∈(0,1). The limiting cases α=0, α=1, and α=∞ follow by continuity of $D_{\alpha }$ and $H_{\alpha }$ for all α≥0. □

Define

(15) $$\pi \left(\alpha \right)=\underset{n\to \infty }{lim inf}\frac{H_{\alpha }\left(r_{n}\right)}{n}$$

Lemma 1 shows that it is impossible to achieve perfect $D_{\alpha }$-smoothing if R<1−π(α). A question of interest is whether there exist sequences of codes of R>1−π(α) that achieve perfect $D_{\alpha }$-smoothing. The next theorem shows that this is the case for α∈(1,∞].

**Theorem 3.**  *Let $r={\left(r_{n}\right)}_{n}$ be a sequence of noise kernels and let α∈(1,∞]. Then,*

(16) $$\begin{array}{c} S_{\alpha }^{r}=1-\pi \left(\alpha \right). \end{array}$$

The proof relies on a random coding argument and is given in Appendix B. This result will be used below to characterize the smoothing capacity of the Bernoulli and ball noise operators.

**Remark 2.**  *Equality (16) does not hold in the case α∈[0,1]. From Theorem 4 below, the Bernoulli noise does not satisfy (16) for α∈[0,1). To construct a counterexample for α=1, consider the noise kernel that is almost uniform except for one distinguished point, for instance, $r_{n}\left(x\right)=2^{-(n+1)}$ for x≠0 and $r_{n}\left(0\right)=\frac{1}{2}+\frac{1}{2^{n+1}}.$ Performing the calculations, we then obtain that $S_{1}^{r}=1$ while $\pi \left(1\right)=\frac{1}{2}.$*

**Remark 3.**  *It is worth noting that π(α) is a decreasing function of α for 0≤α≤∞.*

## 4. Bernoulli Noise

In this section, we characterize the value $S_{\alpha }^{\beta_{\delta }}$ for a range of values of α. Then, we provide explicit code families that attain the $D_{\alpha }$-smoothing capacities.

As already mentioned, the resolvability for $\beta_{\delta }$ with respect to α-divergence was considered by Yu and Tan [3]. Their results, stated in Corollary 1, yield an expression for $S_{\alpha }^{\beta_{\delta }}$ for α∈[0,2]∪{∞}. The next theorem summarizes the current knowledge about $S_{\alpha }^{\beta_{\delta }},$ where the claims for 2<α<∞ form new results.

**Theorem 4.** 
(17) $$\begin{array}{c} S_{\alpha }^{\beta_{\delta }}=\left\{\begin{array}{cc} 0 & if\alpha =0\\ 1-h(\delta ) & if\alpha \in (0,1]\\ 1-h_{\alpha }\left(\delta \right) & if\alpha \in (1,\infty ]. \end{array}\right. \end{array}$$

**Proof.**  The claims for α∈[0,1] follow from Corollary 1. The results for α=(1,∞] follow from Theorem 3 since $\frac{H_{\alpha }\left(\beta_{\delta }\right)}{n}=h_{\alpha }\left(\delta \right)$. □

Having quantified the smoothing capacities, let us examine the code families with strong smoothing properties. Since the $D_{1}$-smoothing capacity and the Shannon capacity coincide, it is natural to speculate that codes that achieve the Shannon capacity when used on the BSC(δ) would also attain the $D_{1}$-smoothing capacity. However, the following result demonstrates that the capacity-achieving codes do not yield perfect smoothing. For typographical reasons, we abbreviate $T_{\beta_{\delta }}$ by $T_{\delta }$ from this section onward.

**Proposition 2.**  *Let $C_{n}$ be a sequence of codes achieving a capacity of BSC(δ). Then,*

$$D\left(T_{\delta }f_{C_{n}}∥U_{n}\right)\to \infty ,\;D\left(T_{\delta }f_{C_{n}}∥U_{n}\right)=o\left(n\right).$$

**Proof.**  The second part of the statement is Theorem 2 in [46]. The first part is obtained as follows: Let $C_{n}$ be a capacity-achieving sequence of codes in BSC(δ). Then, from [47] (Theorem 49), there exists a constant K>0 such that $nR\left(C_{n}\right)\leq n\left(1-h\left(\delta \right)\right)-K\sqrt{n}$ for large *n*. Therefore,

$$\begin{array}{c} 0\leq H\left(X_{C_{n}}|Y_{\mathrm{BSC}(\delta ),X}\right)=n\left(R\left(C_{n}\right)+h\left(\delta \right)-1\right)+D\left(T_{\delta }f_{C_{n}}∥U_{n}\right), \end{array}$$

which implies $D\left(T_{\delta }f_{C_{n}}∥U_{n}\right)\geq K\sqrt{n}$. □

Apart from random codes, only polar codes are known to achieve $D_{1}$-smoothing capacity. Before stating the formal result, recall that polar codes are formed by applying several iterations of a linear transformation to the input, which results in creating virtual channels for individual bits with Shannon’s capacity close to zero or to one, plus a vanishing proportion of intermediate-capacity channels. While by Proposition 2, that polar codes that achieve the BSC capacity cannot achieve $D_{1}$-smoothing capacity, adding some intermediate-bit channels to the set of data bits makes this possible. This idea was first introduced in [39] and expressed in terms of resolvability in [48].

**Theorem 5**  ([48], Proposition 1). *Let W be the BSC(δ) channel and $W_{n}^{\left(i\right)}$ be the virtual channels formed after applying n steps of the polarization procedure. For γ∈(0,1/2), define $G_{n}=\left\{i\in \left\{1,\ldots ,n\right\}:C\left(W_{n}^{\left(i\right)}\right)\geq 2^{-n^{\gamma }}\right\}$. Let $C_{n}$ be the polar code corresponding to the virtual channels $G_{n}$. Then, $D(T_{\delta }f_{C_{n}}∥U_{n})\to 0$.*

Note that $\lim_{n\to \infty }R\left(C_{n}\right)=\lim_{n\to \infty }\frac{|G_{n}|}{n}=1-h\left(\delta \right)$. Hence, the polar code construction presented above achieves the perfect smoothing threshold with respect to the KL divergence. Furthermore, since the convergence in the α divergence for α<1 is weaker than the convergence in α=1, the same polar code sequence is perfectly $D_{\alpha }$-smoothable for α<1. Noting that the smoothing threshold for α<1 is 1−h(δ) by Theorem 4, we conclude that the above polar code sequence achieves smoothing capacity in α-divergence for α<1.

As mentioned earlier, the smoothing properties of code families other than random codes and polar codes have not been extensively studied. We show that the duals of capacity-achieving codes in the BEC exhibit good smoothing properties using the tools developed in [10]. As the first step, we establish a connection between the smoothing of a generic linear code and the erasure correction performance of its dual code.

**Lemma 2.**  *Let C be a linear code and let $X_{C^{⊥}}$ be a random uniform codeword of $C^{⊥}$. Let $Y_{X_{C^{⊥}},\mathrm{BEC}\left(\lambda \right)}$ be the output of the erasure channel BEC(λ) for the input $X_{C^{⊥}}$. Then,*

(18) $$\begin{array}{c} D_{\alpha }\left(T_{\delta }f_{C}∥U_{n}\right)\leq H\left(X_{C^{⊥}}|Y_{X_{C^{⊥}},\mathrm{BEC}\left(\lambda \right)}\right), \end{array}$$

*where $\lambda ={\left(1-2\delta \right)}^{2}$ for α=1 and $\lambda =1-h_{\alpha }\left(\delta \right)$ for α∈{2,3,…,∞}.*

The proof is given in Appendix D.

Using this lemma, we show that the duals of the BEC capacity-achieving codes (with growing distance) exhibit good smoothing properties. In particular, they achieve $D_{\alpha }$-smoothing capacities for α∈{2,3,…,∞}.

**Theorem 6.**  *Let ${\left(C_{n}\right)}_{n}$ be a sequence of linear codes with rate $R_{n}\to R$. Suppose that the dual sequence ${\left(C_{n}^{⊥}\right)}_{n}$ achieves Shannon’s capacity of the BEC(λ) with λ=R, and assume that $d\left(C_{n}^{⊥}\right)=\omega \left(\log n\right).$ If $R>{\left(1-2\delta \right)}^{2}$, then,*

$$\begin{array}{c} \lim_{n\to \infty }D\left(T_{\delta }f_{C_{n}}∥U_{n}\right)=0. \end{array}$$

*Additionally, for α∈{2,3,…,∞}, if $R>1-h_{\alpha }\left(\delta \right)$, then,*

$$\begin{array}{c} \lim_{n\to \infty }D_{\alpha }\left(T_{\delta }f_{C_{n}}∥U_{n}\right)=0. \end{array}$$

*In particular, the sequence $C_{n}$ achieves $D_{\alpha }$-smoothing capacity $S_{\alpha }^{\beta_{\delta }}$ for α∈{2,3,…,∞}.*

**Proof.**  Since the dual codes achieve the capacity of the BEC, it follows from ([49], Theorem 5.2) that, if their distance grows with *n*, then their decoding error probability vanishes. In particular, if $d\left(C_{n}^{⊥}\right)=\omega \left(\log\left(n\right)\right),$ then, $P_{B}\left(\mathrm{BEC}\left(R-\epsilon \right),C_{n}^{⊥}\right)=o\left(\frac{1}{n}\right)$ for all ϵ∈(0,R]. Hence, from Fano’s inequality,

$$\lim_{n\to \infty }H\left(X_{C_{n}^{⊥}}|Y_{X_{C_{n}^{⊥}},\mathrm{BEC}\left(R-\epsilon \right)}\right)=0.$$

Now, if $R>{\left(1-2\delta \right)}^{2}$, then there exists $\epsilon_{0}$ such that $R-\epsilon_{0}={\left(1-2\delta \right)}^{2}$. Therefore, from Lemma 2,

$$\begin{array}{c} \lim_{n\to \infty }D\left(T_{\delta }f_{C_{n}}∥U_{n}\right)\leq \lim_{n\to \infty }H\left(X_{C_{n}^{⊥}}|Y_{X_{C_{n}^{⊥}},\mathrm{BEC}\left(R-\epsilon_{0}\right)}\right)=0. \end{array}$$

Similarly, $\;\mathrm{if}\;R>1-h_{\alpha }\left(\delta \right)\;\mathrm{for}\;\alpha \in \left\{2,3,\ldots ,\infty \right\},\;\mathrm{then},\;\lim_{n\to \infty }D_{\alpha }\left(T_{\delta }f_{C_{n}}∥U_{n}\right)=0.$

Together with Theorem 4, we have now proved the final claim. □

The known code families that achieve the capacity of the BEC include polar codes, LDPC codes, and doubly transitive codes, such as constant-rate RM codes. LDPC codes do not fit the assumptions because of low dual distance, but the other codes do. This yields explicit families of codes that achieve the $D_{\alpha }$-smoothing capacity.

**Figure 1:**
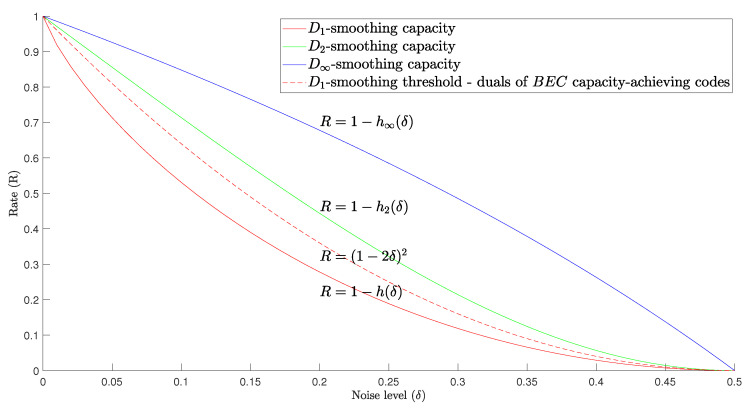
Capacities and achievable rates for perfect smoothing. The lowermost curve gives the Shannon capacity of the BSC(δ), the second curve from the bottom is the smoothing threshold for the duals of the BEC capacity-achieving codes, the third one is $S_{2}^{\beta_{\delta }}$ and the top one is $S_{\infty }^{\beta_{\delta }}$.

We illustrate the results of this section in Figure 1, where the curves show the achievability and impossibility rates for perfect smoothing with respect to the Bernoulli noise. Given a code (sequence) of rate *R*, putting it through a noise $\beta_{\delta }$ below the Shannon capacity cannot achieve perfect smoothing. The sequence of polar codes from [39], cited in Theorem 5, is smoothable at rates equal to the Shannon capacity, although these codes do not provide a decoding guarantee at that noise level. At the second curve from the bottom, the duals of the codes that achieve Shannon’s capacity in BEC achieve perfect $D_{1}$-smoothing; at the third (fourth) curve, these codes are perfectly $D_{2}$- (or $D_{\infty }$-) smoothable, and they achieve the corresponding smoothing capacity.

**Remark 4.**  *Observe that the strong converse of the channel coding theorem does not imply perfect smoothing. To give a quick example, consider a code $C_{n}=B\left(0,\delta^{\prime }n\right)$ formed of all the vectors in the ball. Let 0<δ<1/2 and let us use this code on a BSC(δ), where $h\left(\delta \right)+h\left(\delta^{\prime }\right)>1$ and δ<1/2. From the choice of the parameters, the rate of $C_{n}$ is above capacity, and, therefore, $P_{B}\left(\mathrm{BSC}\left(\delta \right),C_{n}\right)\approx 1$ from the strong converse. At the same time,*

$$\begin{array}{cc} D(T_{\delta }f_{C_{n}}∥U_{n}) & =n-H\left(b_{\delta^{\prime }n}*\beta_{\delta }\right)=n-H\left(\beta_{\delta^{\prime }}*\beta_{\delta }\right)+O\left(\sqrt{n}\right)\\ \; & =n\left(1-h\left(\delta^{\prime }\left(1-\delta \right)+\delta \left(1-\delta^{\prime }\right)\right)\right)+O\left(\sqrt{n}\right). \end{array}$$

*where the transition from the ball noise to the Bernoulli noise (the second equality) is shown in [30]. Since $\delta^{\prime }\left(1-\delta \right)+\delta \left(1-\delta^{\prime }\right))<1/2$ for all $\delta <1/2,\delta^{\prime }<1/2,$ we conclude that $D(T_{\delta }f_{C_{n}}∥U_{n})↛0.$*

**Remark 5.**  *In this paper, we mostly study the trade-off between the rate of codes and the level of the noise needed to achieve perfect smoothing. A recent work of Debris-Alazard et al. [4] considered guarantees for smoothing derived from the distance distribution of codes and their dual distance (earlier, similar calculations were performed in [42,50]). Our approach enables us to find the conditions for perfect smoothing similar to [4] but relying on fewer assumptions.*

**Proposition 3.**  *Let $C_{n}$ be a sequence of codes whose dual distance $d\left(C_{n}^{⊥}\right)\geq \partial^{⊥}n$ where $\partial^{⊥}\in \left(0,1\right)$. If $\partial^{⊥}>{\left(1-2\delta \right)}^{2}$, then,*

$$\begin{array}{c} \lim_{n\to \infty }D\left(T_{\delta }f_{C_{n}}∥U_{n}\right)=0. \end{array}$$

**Proof.**  *Notice that $\lim_{n\to \infty }H\left(X_{C_{n}^{⊥}}|Y_{X_{C^{⊥}},\mathrm{BEC}\left(\lambda \right)}\right)=0$ if $\partial^{⊥}>\lambda$. With this, the proof is a straightforward application of Lemma 2. □*

Compared to [4], this claim removes the restrictions on the support of the dual distance distribution of the codes $C_{n}.$

## 5. Binary Symmetric Wiretap Channels

In this section, we discuss applications of perfect smoothing to the BSC wiretap channel. Wyner’s wiretap channel model V [35] for the case of BSCs is defined as follows: The system is formed of three terminals, A,B, and *E*. Terminal *A* communicates with *B* by sending messages *M* chosen from a finite set M. Communication from *A* to *B* occurs over a BSC $W_{b}$ with crossover probability $\delta_{b}$, and it is observed by the eavesdropper *E* via another BSC $W_{e}$ with crossover probability $\delta_{e}>\delta_{b}.$ A message M∈M is encoded into a bit sequence $X\in H_{n}$ and sent from *A* to *B* in *n* uses of the channel $W_{b}.$ Terminal *B* observes the sequence $Y=X+W_{b},$ where $W_{b}∼\mathrm{Bin}\left(n,\delta_{b}\right)$ is the noise vector, while terminal *E* observes the sequence $Z=X+W_{e}$ with $W_{e}∼\mathrm{Bin}\left(n,\delta_{e}\right)$. We assume that the messages are encoded into a subset of $H_{n},$ which imposes some probability distribution on the input of the channels. The goal of the encoding is to ensure reliability and secrecy of communication. The reliability requirement amounts to the condition $\mathrm{Pr}(M\neq \hat{M})\to 0$ as n→∞, where $\hat{M}$ is the estimate of *M* made by *B*. To ensure secrecy, we require the *strong secrecy condition* I(M;Z)→0. This is in contrast to the condition $\frac{1}{n}I\left(M;Z\right)\to 0$ studied in the early works on the wiretap channel, which is now called weak secrecy. Denote by $R=\frac{1}{n}\log\left|M\right|$ the transmission rate. The *secrecy capacity*$C_{s}\left(V\right)$ is defined as the supremum of the rates that permit reliable transmission, which also conforms to the secrecy condition.

The nested coding scheme, proposed by Wyner [35], has been the principal tool of constructing well-performing transmission protocols for the wiretap channel [38,39,41]. To describe it, let $C_{e}$ and $C_{b}$ be two linear codes such that $C_{e}\subset C_{b}$ and $\left|M\right|=\frac{|C_{b}|}{|C_{e}|}$. We assign each message *m* to a unique coset of $C_{e}$ in $C_{b}$. The sequence transmitted by *A* is a uniform random vector from the coset. As long as the rate of the code $C_{b}$ is below the capacity of $W_{b}$, we can ensure the reliability of communication from *A* to *B*.

Strong secrecy can be achieved relying on perfect smoothing. Denote by $c_{m}$ a leader of the coset that corresponds to the message *m*. The basic idea is that if $P_{Z|M=m}=\left(T_{\delta }f_{C_{e}}\right)\left(\cdot +c_{m}\right)$ is close to a uniform distribution $U_{n}$ for all *m*, these conditional pmfs are almost indistinguishable from each other, and terminal *E* has no means of inferring the transmitted message from the observed bit string *Z*.

As mentioned earlier, the weak secrecy results for the wiretap channel based on LDPC codes and on polar codes were presented in [38,39], respectively. The problem that these schemes faced, highlighted in Theorems 2 and 5, is that code sequences that achieve BSC capacity have a rate gap of at least $1/\sqrt{n}$ to the capacity value. At the same time, the rate of perfectly smoothable codes must exceed the capacity by a similar quantity [51]. For this reason, the authors of [39] included the intermediate virtual channels in their polar coding scheme, which gave them strong secrecy, but interfered with transmission reliability. A similar general issue arose earlier in attempting to use LDPC codes for the wiretap channel [40].

Contributing to the line of work connecting smoothing and thewiretap channel [2,3,11], we show that nested coding schemes $C_{e}\subset C_{b},$ where the code $C_{b}$ is good for error correction in $\mathrm{BSC}(\delta_{b})$ and $C_{e}$ is perfectly smoothable with respect to $\beta_{\delta_{b}}$, attain strong secrecy and reliability for a BSC wiretap channel $\left(\delta_{b},\delta_{e}\right)$. As observed in Lemma 2, the duals of the good erasure-correcting codes are perfectly smoothable for certain noise levels and, hence, they form a good choice for $C_{e}$ in this scenario.

The following lemma establishes a connection between the smoothness of a noisy distribution of a code and strong secrecy.

**Lemma 3.**  *Consider the nested coding scheme for the BSC wiretap channel introduced above. If $D(T_{\delta_{e}}f_{C_{e}}∥U_{n})<\epsilon ,$ then I(M;Z)<ϵ.*

**Proof.**  We have

$$\begin{array}{cc} D(P_{Z|M}∥U_{n}|P_{M}) & =\sum_{\begin{array}{c} m\in M\\ z\in H_{n} \end{array}}P_{MZ}\left(m,z\right)\log\frac{P_{Z|M}\left(z|m\right)}{U_{n}\left(z\right)}\\ \; & =I\left(M;Z\right)+D\left(P_{Z}∥U_{n}\right). \end{array}$$

Now, note that $P_{Z|M=m}\left(z\right)=\left(T_{\delta_{e}}f_{C_{e}}\right)\left(z+c_{m}\right)=P_{Z|M=m^{\prime }}\left(z+c_{m^{\prime }}+c_{m}\right),$ so $D(P_{Z|M=m}∥U_{n})$ is independent of *m*. Therefore, for all m∈M

$$\begin{array}{cc} D(P_{Z|M=m}∥U_{n}) & =D(P_{Z|M}∥U_{n}|P_{M})\\ \; & =I\left(M;Z\right)+D\left(P_{Z}∥U_{n}\right)\geq I\left(M;Z\right).\;\;□ \end{array}$$

This lemma enables us to formulate conditions for reliable communication while guaranteeing the strong secrecy condition. Namely, it suffices to take a pair (a sequence of pairs) of nested codes $C_{e}\subset C_{b}$ such that $D(T_{\delta_{e}}f_{C_{e}}∥U_{n})\to 0$ as n→∞. If at the same time the code $C_{b}$ corrects errors on a BSC$(\delta_{b}),$ then the scheme fulfills both the reliability and strong secrecy requirements under noise levels $\delta_{b}$ and $\delta_{e}$ for channels $W_{b}$ and $W_{e}$, respectively, supporting transmission from *A* to *B* at rate $R_{b}-R_{e}$. Together with the results established earlier, we can now make this claim more specific.

**Theorem 7.**  *Let ${\left({\left(C_{e}^{n}\right)}^{⊥}\right)}_{n}$ and ${\left(C_{b}^{n}\right)}_{n}$ be sequences of linear codes that achieve the capacity of the BEC for their respective rates. Suppose that $C_{e}^{n}\subset C_{b}^{n}$ and*

*1*  *$d\left({\left(C_{e}^{n}\right)}^{⊥}\right)=\omega \left(\log n\right),R\left(C_{e}^{n}\right)\to R_{e}$;*
*2*  *$d\left(C_{b}^{n}\right)=\omega \left(\log n\right),R\left(C_{b}^{n}\right)\to R_{b}$.*

*If $R_{b}<1-\log\left(1+2\sqrt{\delta_{b}\left(1-\delta_{b}\right)}\right)$ and $R_{e}>4\delta_{e}\left(1-\delta_{e}\right)$, then the nested coding scheme based on $C_{e}^{n}$ and $C_{b}^{n}$ can transmit messages with rate $R_{b}-R_{e}$ from A to B, satisfying the reliability and strong secrecy conditions.*

**Proof.**  From Corollary A1, the conditions $d\left(C_{b}^{\left(n\right)}\right)=\omega \left(\log n\right)$ and $R_{b}<1-\log\left(1+2\sqrt{\delta_{b}\left(1-\delta_{b}\right)}\right)$ guarantee transmission reliability. Furthermore, by Theorem 6, the conditions $d\left({\left(C_{e}^{n}\right)}^{⊥}\right)=\omega \left(\log n\right)$ and $R_{e}>4\delta_{e}\left(1-\delta_{e}\right)$ imply that $D(T_{\delta_{e}}f_{C_{e}}∥U_{n})\to 0$, which in its turn implies strong secrecy by Lemma 3. □

To give an example of a code family that satisfies the assumptions of this theorem, consider the RM codes of constant rate. Namely, let $C_{e}^{n}\subset C_{b}^{n}$ be two sequences of RM codes whose rates converge to $R_{e}$ and $R_{b}$, respectively. Note that the duals of the RM codes are themselves RM codes. By a well-known result [52], the RM codes achieve the capacity of the BEC, and for any sequence of constant-rate RM codes, the distance scales as $2^{\Theta (\sqrt{n})}$. Therefore, the RM codes satisfy the assumptions of Theorem 7.

Note that for the RM codes, we can obtain a stronger result, based on their error correction properties on the BSC. Involving this additional argument brings them closer to the secrecy capacity under the strong secrecy assumption.

**Theorem 8.**  *Let $C_{e}^{n}$ and $C_{b}^{n}$ be two sequences of RM codes satisfying $C_{e}^{n}\subset C_{b}^{n}$ whose rates approach $R_{e}>0$ and $R_{b}>0,$ respectively. If $R_{b}<1-h\left(\delta_{b}\right)$ and $R_{e}>4\delta_{e}\left(1-\delta_{e}\right)$, then the nested coding scheme based on $C_{e}^{n}$ and $C_{b}^{n}$ supports transmission on a BSC wiretap channel $\left(\delta_{b},\delta_{e}\right)$ with rate $R_{b}-R_{e},$ guaranteeing communication reliability and strong secrecy.*

**Proof.**  Very recently, Abbe and Sandon [53], building upon the work of Reeves and Pfister [54], proved that RM codes achieve capacity in symmetric channels. Therefore, the condition $R_{b}<1-h\left(\delta_{b}\right)$ guarantees reliability. The rest of the proof is similar to that of Theorem 7. □

**Figure 2:**
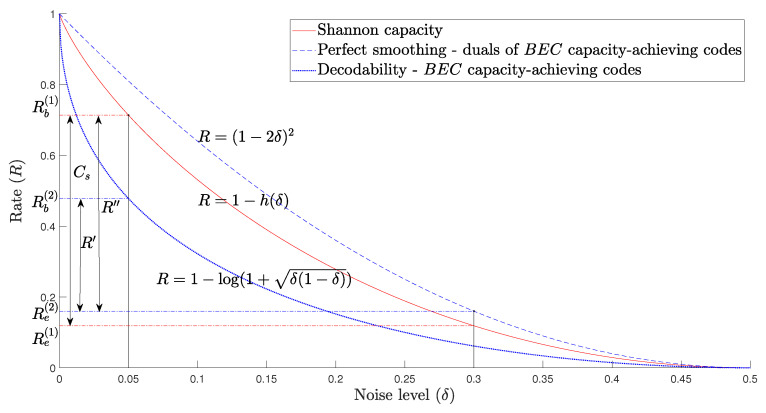
Achievable rates in the BSC wiretap channel with BEC capacity-achieving codes. The bottom curve is the lower bound on the code rate that guarantees decodability on a BSC(δ). The middle curve shows Shannon’s capacity and the top one is the $D_{1}$-smoothing threshold for the Bernoulli noise $T_{\delta }$.

Theorems 7 and 8 stop short of constructing codes that attain the secrecy capacity of the channel (this is similar to the results of [14] for the transmission problem over the BSC). To quantify the gap to capacity, we plot the smoothing and decodability rate bounds in Figure 2.

As an example, let us set the noise parameters $\delta_{b}=0.05$ and $\delta_{e}=0.3$ and denote the corresponding secrecy capacity by $C_{s}$. Suppose that we use a BEC capacity-achieving code as code $C_{b}$ and a dual of a BEC capacity-achieving code as code $C_{e}$ in the nested scheme. The value $R^{\prime }$ is the largest rate at which we can guarantee both reliability and strong secrecy. In the example in Figure 2, $C_{s}=R_{b}^{\left(1\right)}-R_{e}^{\left(1\right)}=0.5949$ and $R^{\prime }=R_{b}^{\left(2\right)}-R_{e}^{\left(2\right)}=0.3181$. The only assumption required here is that the codes $C_{e}^{⊥}$ and $C_{b}$ have good erasure correction properties.

As noted, generally, the RM codes support a higher communication rate than the $R^{\prime }$. Let $R^{\prime \prime }$ be their achievable rate. For the same noise parameters as above, we obtain $R^{\prime \prime }=R_{b}^{\left(1\right)}-R_{e}^{\left(2\right)}=0.5536,$ which is closer to $C_{s}$ than $R^{\prime }$.

**Remark 6.**  *The fact that the RM codes achieve capacity in symmetric channels immediately implies that nested RM codes achieve the secrecy capacity in the BSC wiretap channel under weak secrecy. While it is tempting to assume that, coupled with the channel duality theorems of [55,56], this result also implies that RM codes fulfil the strong secrecy requirement on the BSC wiretap channel, an immediate proof looks out of reach [57].*

### Secrecy from α-Divergence

Classically, the (strong) secrecy in the wiretap channel is measured by I(M,Z). In [11], slightly weaker secrecy measures were considered besides the mutual information. However, more stringent secrecy measures may be required in certain scenarios; α-divergence-based secrecy measures were introduced by Yu and Tan [3] as a solution to this problem.

Observe that the secrecy measured by $D_{\alpha }(P_{Z|M}∥U_{n}\left|M\right)$ for α≥1 is stronger than the mutual-information-based secrecy. This is because for α≥1

$$\begin{array}{c} I\left(M;Z\right)\leq D(P_{Z|M}∥U_{n}|P_{M})\leq D_{\alpha }(P_{Z|M}∥U_{n}\left|P_{M}\right). \end{array}$$

Given a wiretap channel with an encoding-decoding scheme, we say the α-secrecy is satisfied if

$$\begin{array}{c} \lim_{n\to \infty }D_{\alpha }(P_{Z|M}∥U_{n}\left|P_{M}\right)=0. \end{array}$$

The following theorem establishes that it is possible to achieve the rate $C\left(\delta_{b}\right)-S_{\alpha }^{\beta_{\delta_{e}}}=h_{\alpha }\left(\delta_{e}\right)-h\left(\delta_{b}\right)$ with RM codes for α∈{2,3,…,∞}.

**Theorem 9.**  *Let α∈{2,3,…,∞}. Let $C_{e}^{n}$ and $C_{b}^{n}$ be two sequences of RM codes satisfying $C_{e}^{n}\subset C_{b}^{n}$ whose rates approach $R_{e}>0$ and $R_{b}>0,$ respectively. If $R_{b}<1-h\left(\delta_{b}\right)$ and $R_{e}>1-h_{\alpha }\left(\delta_{e}\right)$, then the nested coding scheme based on $C_{e}^{n}$ and $C_{b}^{n}$ supports transmission on a BSC wiretap channel $\left(\delta_{b},\delta_{e}\right)$ guaranteeing α-secrecy with rate $R_{b}-R_{e},$ provided that $h_{\alpha }\left(\delta_{e}\right)-h\left(\delta_{b}\right)>0$.*

Evidently, to achieve a stringent version of secrecy, it is necessary to reduce the rate of the message. The capacity of the $\left(\delta_{b},\delta_{e}\right)$-wiretap channel is $h\left(\delta_{e}\right)-h\left(\delta_{b}\right)$, while the known highest rate that assures α-secrecy and reliability is $h_{\alpha }\left(\delta_{e}\right)-h\left(\delta_{b}\right)$. Hence, to achieve α-secrecy, we must give up $h\left(\delta_{e}\right)-h_{\alpha }\left(\delta_{e}\right)$ of the attainable rate.

## 6. Ball Noise and Error Probability of Decoding

This section focuses on achieving the best possible smoothing with respect to the ball noise. As an application, we show that codes that possess good smoothing properties with respect to the ball noise are suitable for error correction in the BSC.

### 6.1. Ball Noise

Recall that the perfect smoothing of a sequence of codes is only possible if the rate is greater than the corresponding $D_{\alpha }$-smoothing capacity. In addition to characterizing the $D_{\alpha }$-smoothing capacities of the ball noise, we quantify the best smoothing one can expect with rates below the $D_{\alpha }$-smoothing capacity. We will use these results in the upcoming subsection when we derive upper bounds for the decoding error probability on a BSC. The next theorem summarizes our main result on smoothing with respect to the ball noise.

**Theorem 10.**  *Let ${\left(b_{\delta n}\right)}_{n}$ be the sequence of ball noise operators, where δn is the radius of the ball. Let δ∈[0,1/2],α∈[0,∞]. Let $C_{n}$ be a code of length n and rate $R_{n}.$ Then, we have the following bounds:*

(19) $$\begin{array}{cc} D_{\alpha }\left(T_{b_{\delta n}}f_{C_{n}}∥U_{n}\right) & \geq 0 \end{array}$$

(20) $$\begin{array}{cc} \frac{1}{n}D_{\alpha }\left(T_{b_{\delta n}}f_{C_{n}}∥U_{n}\right) & \geq 1-R_{n}-h\left(\delta \right). \end{array}$$

*There exist sequences of codes of rate $R_{n}\to R$ that achieve asymptotic equality in (19) for all R>1−h(δ). At the same time, if R<1−h(δ), then there exist sequences of codes achieving asymptotic equality in (20).*

**Proof.**  The inequality in (19) is trivial. Let us prove that asymptotically it can be achieved with equality. From Theorem 3, there exists a sequence of codes ${\left(C_{n}\right)}_{n}$ such that $D_{\infty }\left(T_{b_{\delta n}}f_{C_{n}}∥U_{n}\right)=$o(1) given that R>1−h(δ). Hence, for α∈[0,∞]

$$\begin{array}{c} 0\leq D_{\alpha }\left(T_{b_{\delta n}}f_{C_{n}}∥U_{n}\right)\leq D_{\infty }\left(T_{b_{\delta n}}f_{C_{n}}∥U_{n}\right)=o\left(1\right). \end{array}$$

Hence, the equality case in (19) is achievable for all α∈[0,∞].

Let us prove (20). From Lemma 1, we have

$$\begin{array}{cc} D_{\alpha }\left(T_{b_{\delta n}}f_{C_{n}}∥U_{n}\right) & \geq n\left(1-R_{n}\right)-H_{\alpha }\left(b_{\delta n}\right)\geq n\left(1-R_{n}-h\left(\delta \right)\right) \end{array}$$

because $\frac{1}{n}H_{\alpha }\left(b_{\delta n}\right)=\frac{1}{n}\log V_{\delta n}\leq h\left(\delta \right)$.

We are left to show that for R<1−h(δ), (20) can be achieved with equality in the limit of large *n*. We use a random coding argument to prove this. Let $C_{n}$ be an $\left(n,2^{nR_{n}}\right)$ code whose codewords are chosen independently and uniformly. In Equation (A6), Appendix B, we define the expected norm of the noisy function. Here, we use this quantity for the ball noise kernel. For α∈[0,∞), define

$$Q_{n}\left(\alpha \right)=E_{C_{n}}2^{\left(\alpha -1\right)D_{\alpha }\left(T_{b_{\delta n}}∥U_{n}\right)}.$$

From Lemma A2, for any rational α≥1,

(21) Qn(α)≤∑k=0ppk2nkq(1−Rn−logVδnn)Qnp−kq,

for $p,q\in Z_{0}^{+}$ such that $\alpha =1+\frac{p}{q}$.

Assume that R<1−h(δ). Let us prove that $Q_{n}\left(\alpha \right)\leq 2^{n(\alpha -1)(1-R-h(\delta )+o(1))}$ for rational values of α using induction. Let α∈[1,2] be rational and note that p≤q. Since $Q_{n}\left(\cdot \right)\leq 1$ when the argument is less than 1, we can write (21) as follows:

Qn(α)≤∑k=0ppk2nkq(1−Rn−logVδnn)=2n(α−1)(1−R−h(δ)+o(1)).

Now, assume that (21) holds for all rational α∈[1,m] for some integer m≥2 and prove that, in this case, it holds also for α∈(m,m+1]. By the induction hypothesis,

Qn(α)≤∑0≤k≤p−qpk2nkq(1−Rn−logVδnn)2np−k−qq(1−R−h(δ)+o(1))+∑k=p−qppk2nkq(1−Rn−logVδnn)≤∑0≤k≤p−qpk2n(α−2)(1−R−h(δ)+o(1))+∑k=p−qppk2n(α−1)(1−R−h(δ)+o(1))=2n(α−1)(1−R−h(δ)+o(1)).

Therefore, for every rational α∈(1,∞), there exists a sequence of codes satisfying

(22) $$\begin{array}{c} D_{\alpha }\left(T_{b_{\delta n}}f_{C_{n}}∥U_{n}\right)=n\left(1-R-h\left(\delta \right)+o\left(1\right)\right), \end{array}$$

which is equivalent to the equality in (20).

Let us extend this result to non-negative reals. Let α∈[0,∞) and let us choose a rational $\alpha^{\prime }\in \left(1,\infty \right)$ such that $\alpha <\alpha^{\prime }$. We know that there exists a sequence of codes satisfying

$$\begin{array}{c} D_{\alpha^{\prime }}\left(T_{b_{\delta n}}f_{C_{n}}∥U_{n}\right)=n\left(1-R-h\left(\delta \right)+o\left(1\right)\right). \end{array}$$

From (20) and from Remark 1,

$$\begin{array}{c} n\left(1-R_{n}-h\left(\delta \right)\right)\leq D_{\alpha }\left(T_{b_{\delta n}}f_{C_{n}}∥U_{n}\right)\leq D_{\alpha^{\prime }}\left(T_{b_{\delta n}}f_{C_{n}}∥U_{n}\right)=n\left(1-R-h\left(\delta \right)+o\left(1\right)\right). \end{array}$$

Hence, the asymptotic equality in (20) is achievable for all α∈[0,∞). □

The above theorem characterizes the $D_{\alpha }$-smoothing capacities with respect to ball noise.

**Corollary 2.**  *Let δ∈[0,1/2]. Let $b\left(\delta \right)={\left(b_{\delta n}\right)}_{n}$ be a sequence of ball noise operators, where δn is the radius corresponding to the n-th kernel. Then,*

$$\begin{array}{cc} S_{\alpha }^{b(\delta )} & =1-h(\delta )\;\mathrm{for}\;\alpha \in [0,\infty ]. \end{array}$$

The norms of $T_{b_{t}}f_{C}$ can be used to bound the decoding error probability on a BSC. While estimating these norms for a given code is generally complicated, the second norm affords a compact expression based on the distance distribution of the code. In the next section, we bound the decoding error probability using the second norm of $T_{b_{t}}f_{C}$. The following proposition provides closed-form expressions for $∥2^{n}T_{b_{t}}f_{C}{∥}_{2}^{2}$.

**Proposition 4.** 
$$\begin{array}{c} ∥2^{n}T_{b_{t}}f_{C}{∥}_{2}^{2}=\frac{2^{n}}{\left|C\right|V_{t}^{2}}\sum_{i=0}^{n}\mu_{t}\left(i\right)A_{i}=\frac{1}{V_{t}^{2}}\sum_{k=0}^{n}L_{t}{\left(k\right)}^{2}A_{k}^{⊥}. \end{array}$$

*where $\mu_{t}\left(i\right)$ is defined in (1) and $L_{t}$ is the Lloyd polynomial of degree t (A2).*

The proof is immediate from Proposition A1 in combination with (A2) and (A4).

### 6.2. Probability of Decoding Error on a BSC(δ)

The idea that the smoothing of codes under some conditions implies good decoding performance has appeared in a number of papers using different language. The smoothing of capacity-achieving codes was considered in [18,46]. Hązła et al. [14] showed that if a code (sequence) is perfectly smoothable with respect to the Bernoulli noise, then the dual code is good for decoding (see Theorem A4, Corollary A1). Going from smoothing to decodability involves representing the $D_{2}$-smoothness of codes with respect to the Bernoulli noise as a potential energy form and comparing it to the Bhattacharyya bound for the dual codes. One limitation of this approach is that it cannot infer decodability for rates $R>1-\log(1+2\sqrt{\delta (1-\delta )})$ (this is the region above the blue solid curve in Figure 2). Rao and Sprumont [15] and Hązła [34] proved that sufficient smoothing of codes implies the decodability of the codes themselves rather than their duals. However, these results are concerned with list decoding for rates above the Shannon capacity, resulting in an exponential list size, which is arguably less relevant from the perspective of communication.

Except for [15], the cited papers utilize perfect or near-perfect smoothing to infer decodability. For codes whose rates are below the capacity, perfect smoothing is impossible. At the same time, codes that possess sufficiently good smoothing properties are good for decoding. This property is at the root of the results for list decoding in [15]; however, their bounds were insufficient to make conclusions about list decoding below capacity.

Consider a channel where, for the input $X∼f_{C}$, the output *Y* is given by Y=X+W with $W∼b_{t}$. Define $F_{t}\left(y\right)=\left|C\cap B\left(y,t\right)\right|$ as the number of codewords in the ball B(y,t). Hence, for a received vector *y*, the possible number of codewords that can yield *y* is given by $F_{t}\left(y\right)$. Intuitively, the decoding error is small if $F_{t}\left(y\right)\approx 1$ for typical errors. Therefore, $F_{t}$ is of paramount interest in decoding problems. Since the typical errors for both ball noise and the Bernoulli noise are almost the same, this allows us to obtain a bound for decodability in the BSC channel. Using this approach, we show that the error probability of decoding on a BSC(δ) can be expressed via the second moment of the number of codewords in the ball of radius t≳δn.

Assume, without loss of generality, that C is a linear code and $0^{n}$ is used for transmission. Let *Y* be the random Bernoulli vector of errors, and note that $Y∼\beta_{\delta }$. The calculation below does not depend on whether we rely on unique or list decoding within a ball of radius *t*, so let us assume that the decoder outputs L≥1 candidate codewords conditioned on the received vector *y*, which is a realization of Y.

In this case, the list decoding error can be written as

(23) $$\begin{array}{c} P_{L,t}\left(C,\mathrm{BSC}\left(\delta \right)\right)=\mathrm{Pr}\{F_{t}\left(Y\right)\geq L+1\cup |Y|>t\}. \end{array}$$

**Theorem 11.**  *Let t and $t^{\prime }$ be integers such that $0<t^{\prime }<t<n$. Then, for any L≥1,*

(24) $$\begin{array}{c} P_{L,t}\left(C,\mathrm{BSC}\left(\delta \right)\right)\leq \frac{\beta_{\delta }\left(t^{\prime }\right)}{L}\sum_{w=1}^{n}\mu_{t}\left(w\right)A_{w}+\mathrm{Pr}(|Y|\leq t^{\prime }\cup |Y|\geq t). \end{array}$$

**Proof.**  Define $S_{t^{\prime },t}=B\left(0,t\right)\setminus B\left(0,t^{\prime }\right)$. Clearly,

$$\begin{array}{cc} P_{L,t}\left(C,\mathrm{BSC}\left(\delta \right)\right) & =\mathrm{Pr}\{F_{t}\left(Y\right)\geq L+1\cup |Y|>t\}\\ \; & \leq \mathrm{Pr}\left\{\left(F_{t}\left(Y\right)\geq L+1\right)\cap \left(Y\in S_{t^{\prime },t}\right)\right\}+\mathrm{Pr}\left(Y\notin S_{t^{\prime },t}\right). \end{array}$$

Let us estimate the first of these probabilities.

$$\begin{array}{cc} \; & \mathrm{Pr}\{\left(F_{t}\left(Y\right)\geq L+1\right)\cap \left(Y\in S_{t^{\prime },t}\right)\}\\ \; & =\sum_{y\in S_{t^{\prime },t}}1_{F_{t}\left(y\right)\geq L+1}\beta_{\delta }\left(y\right)\\ \; & \leq \sum_{y\in S_{t^{\prime },t}}\frac{F_{t}\left(y\right)-1}{L}\beta_{\delta }\left(y\right)\\ \; & \leq \frac{\beta_{\delta }\left(t^{\prime }\right)}{L}\sum_{y\in S_{t^{\prime },t}}\left(F_{t}\left(y\right)-1\right)\\ \; & \leq \frac{\beta_{\delta }\left(t^{\prime }\right)}{L}\sum_{y\in B(0,t)}\left(F_{t}\left(y\right)-1\right)\;(\mathrm{because}\;\mathrm{for}\;\mathrm{all}\;y\in B\left(0,t\right),F_{t}\left(y\right)\geq 1)\\ \; & =\frac{\beta_{\delta }\left(t^{\prime }\right)}{L}\left(\sum_{y\in H_{n}}\left(1_{C}*1_{B(0,t)}\right)\left(y\right)1_{B(0,t)}\left(y\right)-V_{t}\right)\\ \; & =\frac{\beta_{\delta }\left(t^{\prime }\right)}{L}\left(\sum_{c\in C}\left(1_{B(0,t)}*1_{B(0,t)}\right)\left(c\right)-V_{t}\right)\\ \; & =\frac{\beta_{\delta }\left(t^{\prime }\right)}{L}\sum_{i=1}^{n}\mu_{t}\left(i\right)A_{i}.\;\;□ \end{array}$$

**Remark 7.**  *In the case of L=1, the bound in (24) can be considered a slightly weaker version of Poltyrev’s bound [58], Lemma 1. By allowing this weakening, we obtain a bound in a somewhat more closed form, also connecting the decodability with smoothing. We also prove a simple bound for the error probability of list decoding expressed in terms of the code’s distance distribution (and, from (A4), also in terms of the dual distance distribution). The latter result seems not to have appeared in earlier literature.*

The following version of this lemma provides an error bound, which is useful in the asymptotic setting.

**Proposition 5.**  *Let $t=\delta n+n^{\theta },$ where θ∈(1/2,1). Then,*

$$\begin{array}{c} P_{L,t}\left(C,\mathrm{BSC}\left(\delta \right)\right)\leq \frac{\sqrt{2n}}{LV_{t}}{\left(\frac{1-\delta }{\delta }\right)}^{2n^{\theta }}\sum_{w=1}^{n}\mu_{t}\left(w\right)A_{w}+2e^{-n^{2\theta -1}}. \end{array}$$

*In particular,*

$$\begin{array}{c} P_{L,t}\left(C,\mathrm{BSC}\left(\delta \right)\right)\leq \frac{\sqrt{2n}}{V_{t}}{\left(\frac{1-\delta }{\delta }\right)}^{2n^{\theta }}\sum_{w=1}^{n}\mu_{t}\left(w\right)A_{w}+2e^{-n^{2\theta -1}}. \end{array}$$

**Proof.**  Set $t^{\prime }=\delta n-n^{\theta }$. A direct calculation shows that

$$\beta_{\delta }\left(t^{\prime }\right)V_{t}<\sqrt{2n}{\left(\frac{1-\delta }{\delta }\right)}^{2n^{\theta }}.$$

By the Hoeffding bound,

$$\begin{array}{c} \mathrm{Pr}(|Y|\leq t^{\prime }\cup |Y|\geq t)\leq 2e^{-n^{2\theta -1}}. \end{array}$$

Together with Lemma 11, this implies our statements. □

A question of prime importance is whether the right-hand side quantities in Proposition 5 converge to 0. For R<1−h(δ), one can easily see that for random codes, $\sum_{w=1}^{n}\frac{\mu_{t}\left(w\right)}{V_{t}}A_{w}=2^{-\Theta (n)}$, where $t=\delta n+n^{\theta }$, showing that this is, in fact, the case.

From Proposition 4, it is clear that the potential energy $\sum_{w=1}^{n}\mu_{t}\left(w\right)A_{w}$ is a measure of the smoothness of $T_{b_{t}}f_{C}$. This implies that codes that are sufficiently smoothable with respect to $b_{t}$ are decodable in the BSC with vanishing error probability. In other words, Proposition 5 establishes a connection between the smoothing and the decoding error probability.

## 7. Perfect Smoothing—The Finite Case

In this section, we briefly overview another form of perfect smoothing, which is historically the earliest application of these ideas in coding theory. It is not immediately related to the information-theoretic problems considered in the other parts.

We are interested in radial kernels that yield perfect smoothing for a given code. We often write r(i) instead of r(x) if |x|=i, and call ρ(r):=max(i:r(i)≠0) the *radius* of *r*. Note that the logarithm of the support size of *r* (as a function on the space $H_{n}$) is exactly the 0-Rényi entropy of *r*. Therefore, kernels with smaller radii can be perceived as less random, supporting the view of the radius ρ(r) as a general measure of randomness.

**Definition 4.**  *We say a code C is perfectly smoothable with respect to r if $T_{r}f_{C}\left(x\right)=\frac{1}{2^{n}}$ for all $x\in H_{n}$, and, in this case, we say that r is a perfectly smoothing kernel for C.*

Intuitively, such a kernel should have a sufficiently large radius. In particular, it should be as large as the *covering radius* of the code ρ(C) or otherwise smoothing does not affect the vectors that are ρ away from the code. To obtain a stronger condition, recall that the external distance of code C is $\bar{d}\left(C\right)=\left|\left\{i\geq 1:A_{i}^{⊥}\neq 0\right\}\right|.$

**Proposition 6.**  *Let r be a perfectly smoothing kernel of code C. Then, $\rho \left(r\right)\geq \bar{d}\left(C\right).$*

**Proof.**  Note that perfect smoothing of C with respect to *r* is equivalent to

$$\begin{array}{c} ∥2^{n}T_{r}f_{C}{∥}_{2}^{2}=1, \end{array}$$

which by Proposition A1 is equivalent to the following condition:

$$\begin{array}{c} \sum_{i=1}^{n}\hat{r}{\left(i\right)}^{2}A_{i}^{⊥}=0. \end{array}$$

Therefore,

$$\begin{array}{c} \bar{d}\left(C\right)=|\left\{i\geq 1:A_{i}^{⊥}\neq 0\right\}\left|\leq n-\right|\left\{i\geq 1:\hat{r}\left(i\right)\neq 0\right\}|. \end{array}$$

By definition,

$$\begin{array}{c} \hat{r}=\frac{1}{2^{n}}K^{⊺}r, \end{array}$$

where $K={\left(K_{i}\left(j\right)\right)}_{i,j=0}^{n}$ is the Krawtchouk matrix. Define $I_{1}=\left\{j\in \left\{1,2,\ldots ,n\right\}:\hat{r}\left(j\right)=0\right\}$ and $I_{2}=\left\{i\in \left\{1,2,\ldots ,n\right\}:r\left(i\right)\neq 0\right\}$ then,

$$\begin{array}{c} 0=\hat{r}{|}_{I_{1}}=\frac{1}{2^{n}}K^{⊺}{|}_{\left(I_{1},:\right)}r=\frac{1}{2^{n}}K^{⊺}{{|}_{\left(I_{1},I_{2}\right)}r|}_{I_{2}}. \end{array}$$

This relation implies that there exists a linear combination of Krawtchouk polynomials of degree at most ρ(r) with $|I_{1}|$ roots. Therefore, $\bar{d}\left(C\right)\leq n-|\mathrm{supp}\left({\left\{\hat{r}\left(i\right)\right\}}_{i=1}^{n}\right)\left|=\right|I_{1}|\leq \rho \left(r\right).$ □

Since $\rho \left(C\right)\leq \bar{d}\left(C\right)$, this inequality strengthens the obvious condition ρ(r)≥ρ(C). At the same time, there are codes that are perfectly smoothable by a radial kernel *r* such that ρ(r)=ρ(C).

**Definition 5**  ([59]). *A code C is uniformly packed in the wide sense if there exists rational numbers ${\left\{\alpha_{i}\right\}}_{i=0}^{\rho }$ such that*

$$\begin{array}{c} \sum_{i=0}^{\rho (C)}\alpha_{i}A_{i}\left(x\right)=1\;\;\mathrm{for}\;\mathrm{all}\;x\in H_{n}, \end{array}$$

*where $A_{i}\left(x\right)$ is the weight distribution of the code C−x.*

Our main observation here is that some uniformly packed codes are perfectly smoothable with respect to noise kernels that are minimal in a sense. The following proposition states this more precisely.

**Proposition 7.**  *Let C be a code that is perfectly smoothable by a radial kernel of radius ρ(r)=ρ(C). Then, C is uniformly packed in the wide sense with $\alpha_{i}\geq 0$ for all i.*

**Proof.**  By definition, if C is perfectly smoothable with respect to *r*, then $2^{n}T_{r}f_{C}=1$, which is tantamount to $\sum_{y\in H_{n}}\frac{2^{n}}{\left|C\right|}r\left(y\right)1_{C}\left(x-y\right)=1$ for all $x\in H_{n}$. This condition can be written as $\sum_{i=0}^{\rho }\left(\frac{2^{n}}{\left|C\right|}r\left(i\right)\right)A_{i}\left(x\right)=1$ for all $x\in H_{n}$, completing the proof. □

To illustrate this claim, we list several families of uniformly packed codes ([59,60,61]) that are perfectly smoothable by a kernel of radius equal to the covering radius of the code.

(i) Perfect codes: $r=b_{\rho }$, where ρ=ρ(C) is the covering radius.
(ii) 2-error-correcting BCH codes of length $2^{2m+1},m\geq 2$. The smoothing kernel *r* is given by
  $$r\left(0\right)=r\left(1\right)=L,r\left(2\right)=r\left(3\right)=\frac{3L}{n},r\left(i\right)=0,i\geq 4.$$
(iii) Preparata codes. The smoothing kernel *r* is given by
  $$r\left(0\right)=r\left(1\right)=L,r\left(2\right)=r\left(3\right)=\frac{6L}{n-1},r\left(i\right)=0,i\geq 4.$$
(iv) Binary $\left(2^{m}-1,2^{2^{m}-3m+2},7\right)$ Goethals-like codes [60]. The smoothing kernel *r* is given by
  $$r\left(0\right)=r\left(1\right)=L,r\left(2\right)=r\left(3\right)=\frac{65L}{2n},r\left(4\right)=r\left(5\right)=\frac{30L}{n(n-3)},r\left(i\right)=0,i\geq 4.$$

Here, *L* is a generic notation for the normalizing factor. More examples are found in a related class of *completely regular codes* [62].

Definition 5 does not include the condition that $\alpha_{i}\geq 0$, and, in fact, there are codes that are uniformly packed in the wide sense, but some of the $\alpha_{i}$’s are negative, and, thus, they are not smoothable by a noise kernel of radius ρ(C). One such family is the 3-error-correcting binary BCH codes of length $2^{2m+1},m\geq 2$ [60].

## Data Availability

Not applicable.

## Appendix A. *L*_2_ Smoothing

The Fourier transform of a function $f:H_{n}\to R$ is a function on the dual group ${\hat{H}}_{n}$, which we identify with $H_{n}$:

(A1) $$\hat{f}\left(y\right)=\frac{1}{2^{n}}\sum_{x\in H_{n}}f\left(x\right){\left(-1\right)}^{x\cdot y},\;y\in H_{n}.$$

The Fourier transform of the indicator function of the sphere is given by ${\hat{1}}_{S(0,t)}=\frac{1}{2^{n}}K_{t},$ where Kt(x)=Kt(n)(x)=∑j=0t(−1)jxjn−xt−j is a Krawtchouk polynomial of degree *t*. Then, clearly the Fourier transform of the indicator of the ball is

(A2) $${\hat{1}}_{B(0,t)}=\frac{1}{2^{n}}L_{t},$$

where $L_{t}\left(x\right):=\sum_{i=0}^{t}K_{i}\left(x\right)$ is called the Lloyd polynomial ([63], p. 64). The intersection of balls in (1) can be written as $1_{B(0,t)}*1_{B(x,t)},$ which implies the expression ([42], Lemma 4.1)

(A3) $$\mu_{t}\left(i\right)=2^{-n}\sum_{k=0}^{n}L_{t}{\left(k\right)}^{2}K_{k}\left(i\right).$$

Given a code $C\subset H_{n}$, we define the *dual distance distribution* of C as the set of numbers $A_{j}^{⊥}:=\frac{1}{\left|C\right|}\sum_{i=0}^{n}A_{i}K_{j}\left(i\right),$ where ${\left(A_{i}\right)}_{i=0}^{n}$ is the distance distribution of C (2). Note that when C is linear, the set ${\left(A_{j}^{⊥}\right)}_{j=0}^{n}$ coincides with the distance distribution of its dual code $C^{⊥}.$ For a radial potential *V* on $H_{n}$ and a code C, we have

(A4) $$\begin{array}{c} \sum_{i=0}^{n}V\left(i\right)A_{i}=\left|C\right|\sum_{k=0}^{n}\hat{V}\left(k\right)A_{k}^{⊥}. \end{array}$$

The $L_{2}$-smoothness of a noisy code distribution can be written in terms of the distance distribution or of the dual distance distribution.

**Proposition A1.** 
*Let C be a code and r be a noise kernel. Then,*

$$\begin{array}{c} ∥2^{n}T_{r}f_{C}{∥}_{2}^{2}=\frac{2^{n}}{\left|C\right|}\sum_{i=0}^{n}\left(r*r\right)\left(i\right)A_{i}=4^{n}\sum_{k=0}^{n}\hat{r}{\left(k\right)}^{2}A_{k}^{⊥}. \end{array}$$

**Proof.** Let us prove the first equality:

(A5) $$\begin{array}{cc} ∥2^{n}T_{r}f_{C}{∥}_{2}^{2} & =\frac{1}{2^{n}}\sum_{x\in H_{n}}{\left(2^{n}T_{r}f_{C}\left(x\right)\right)}^{2}\\ \; & =\frac{2^{n}}{{\left|C\right|}^{2}}\sum_{x\in H_{n}}\left(r*1_{C}\right){\left(x\right)}^{2}\\ \; & =\frac{2^{n}}{{\left|C\right|}^{2}}\sum_{x\in H_{n}}\sum_{y\in H_{n}}r\left(x-y\right)1_{C}\left(y\right)\sum_{z\in H_{n}}r\left(x-z\right)1_{C}\left(z\right)\\ \; & =\frac{2^{n}}{{\left|C\right|}^{2}}\sum_{y\in C}\sum_{z\in C}\sum_{x\in H_{n}}r\left(x-y\right)r\left(x-z\right)\\ \; & =\frac{2^{n}}{{\left|C\right|}^{2}}\sum_{y\in C}\sum_{z\in C}\left(r*r\right)\left(y-z\right)\\ \; & =\frac{2^{n}}{\left|C\right|}\sum_{i=0}^{n}\left(r*r\right)\left(i\right)A_{i}. \end{array}$$

The second equality is immediate by noticing that $\hat{r*r}=2^{n}{\hat{r}}^{2}$ and using (A4). □

## Appendix B. Proof of Theorem 3

We will first establish Theorem 3 when α is rational, and then use a density argument to extend the proof to all real numbers. The case α=∞ is handled separately at the end of this appendix.

We will use the following technical claim:

**Lemma A1.** 
*Let x and y be two non-negative reals. Further, let p and q be positive integers. Then,*

(x+y)pq≤∑k=0ppkxkqyp−kq.

**Proof.** Clearly ${\left(x+y\right)}^{\frac{1}{q}}\leq x^{\frac{1}{q}}+y^{\frac{1}{q}}$. Therefore,

(x+y)pq≤(x1q+y1q)p=∑k=0ppkxkqyp−kq.  □

For M≥1, let $C=(c_{0},c_{2},\ldots ,c_{M-1})\;\;□$ be a code whose codewords are chosen randomly and independently from $H_{n}.$ For α∈[0,∞), define

(A6) $$Q_{n}\left(\alpha \right)=E_{C}2^{\left(\alpha -1\right)D_{\alpha }\left(T_{r}f_{C}∥U_{n}\right)}.$$

For α>0, $Q_{n}\left(\alpha \right)={∥2^{n}T_{r}f_{C}\left(x\right)∥}_{\alpha }^{\alpha }.$ Clearly $Q_{n}\left(1\right)=1,Q_{n}\left(\alpha \right)\leq 1\;\mathrm{for}\;\alpha \in \left[0,1\right)$, and $Q_{n}\left(\alpha \right)\geq 1$ for α>1.

In the next lemma, we obtain a recursive bound for $Q_{n}$. We will then use an induction argument to show the full result.

**Lemma A2.** 
*Let $\alpha =\frac{p}{q}+1$ and let $C\subset H_{n}$ be a random code of size $M=2^{nR}$. Then,*

(A7)
Qn(α)≤∑k=0ppk2nkq(1−R−1nH1+k/q(r))Qnp−kq.

**Proof.** In the calculation below, we write E for $E_{C}$. Starting with (A6), we obtain

Qn(α)=E12n∑x∈Hn[2n(r∗fC)(x)]α=2n(α−1)E∑x∈Hn∑z∈Cr(x−z)1Mα=2n(α−1)Mα∑x∈HnE∑i=0M−1r(x−ci)α=2n(α−1)Mα∑x∈HnE∑i=0M−1r(x−ci)∑j=0M−1r(x−cj)α−1=2n(α−1)Mα∑x∈HnE∑i=0M−1r(x−ci)r(x−ci)+∑j=0,j≠iM−1r(x−cj)pq≤2n(α−1)Mα∑x∈HnE∑i=0M−1r(x−ci)∑k=0ppkr(x−ci)kq∑j=0,j≠iM−1r(x−cj)p−kq=2n(α−1)Mα∑k=0ppk∑x∈HnE∑i=0M−1r(x−ci)1+kq∑j=0,j≠iM−1r(x−cj)p−kq=2n(α−1)Mα∑k=0ppk∑x∈HnE∑i=0M−1r(x−ci)1+kqE∑j=0,j≠iM−1r(x−cj)p−kq,

where $c_{i},i=1,\ldots ,M$ are random codewords in the code C. Recalling that $Er{\left(x-c_{i}\right)}^{a}={∥r∥}_{a}^{a}$ for any a>0, we continue as follows:

≤2n(α−1)Mα∑k=0ppk∑x∈HnM∥r∥1+k/q1+k/qE∑j=0M−1r(x−cj)p−kq=2n(α−1)Mα−1∑k=0ppk∥r∥1+k/q1+k/qE∑x∈Hn∑j=0M−1r(x−cj)p−kq=2np/qMp/q∑k=0ppk∥r∥1+k/q1+k/qQnp−kqM(p−k)/q2n((p−k)/q−1)=∑k=0ppk2n(1+k/q)Mk/q∥r∥1+k/q1+k/qQnp−kq=∑k=0ppk2nkq1−R−H(1+k/q)(r)nQnp−kq,

where we used (5) and the fact that *r* is a pmf. □

On account of (14), (A6), and Lemma 1, to prove Theorem 3, we need to prove the following:

**Theorem A1.** 
*Consider a sequence of ensembles of random codes of increasing length n and rate $R_{n}\to R.$ If R>1−π(α), where π(α) is given by (15), then,*

(A8)
$$\begin{array}{c} \lim_{n\to \infty }Q_{n}\left(\alpha \right)=1 \end{array}$$

*for all α∈(1,∞).*

We start with the case of rational α.

**Proposition A2.** 
*Let α≥0 be rational. If R>1−π(α), then ${lim sup}_{n}Q_{n}\left(\alpha \right)\leq 1$.*

**Proof.** This statement is true for all 0≤α<1, so also true for all rational α in [0,1). Assume that it holds for all rational α in [0,m), where $m\in Z^{+}$. Let α∈[m,m+1) and choose $p,q\in Z_{0}^{+}$ such that $\alpha =1+\frac{p}{q}$. By Lemma A2,

lim supnQn(α)≤lim supn∑k=0ppk2nkq1−Rn−H1+k/q(rn)nQnp−kq≤∑k=0ppklim supn2nkq1−Rn−H1+k/q(rn)nlim supnQnp−kq.

If R>1−π(α), then evidently, R>1−π(1+k/q) for all k≤p. Therefore,

$$\underset{n\to \infty }{lim sup}2^{\frac{nk}{q}\left(1-R_{n}-\frac{H_{1+k/q}\left(r_{n}\right)}{n}\right)}=0$$

for all k>0. Since $\frac{p}{q}<m,$ by the induction hypothesis, we have ${lim sup}_{n}Q_{n}\left(\frac{p-k}{q}\right)\leq 1$ for k=0,1,…,p. Therefore, all the terms except the one with k=0 vanish, yielding ${lim sup}_{n}Q_{n}\left(\alpha \right)\leq 1$. □

Since $Q_{n}\left(\alpha \right)\geq 1$ for α>1, this proves Theorem A1 for all rational α∈(1,∞).

Finally, let us extend this result to all real α>1. As a first step, let us show that π(α) is continuous.

**Lemma A3.** 
*π(α) is continuous for 1<α<∞.*

**Proof.** From the monotonicity of the Rényi entropies, for $\alpha^{\prime }>\alpha >1$,

$$\begin{array}{cc} 0 & \leq \pi \left(\alpha \right)-\pi \left(\alpha^{\prime }\right)\\ \; & =\underset{n\to \infty }{lim inf}\frac{1}{n}H_{\alpha }\left(r_{n}\right)-\underset{n\to \infty }{lim inf}\frac{1}{n}H_{\alpha^{\prime }}\left(r_{n}\right). \end{array}$$

Now, let us choose a subsequence ${\left(r_{n_{k}}\right)}_{k}$ such that

$$\begin{array}{c} \lim_{k\to \infty }\frac{1}{n_{k}}H_{\alpha^{\prime }}\left(r_{n_{k}}\right)=\underset{n\to \infty }{lim inf}\frac{1}{n}H_{\alpha^{\prime }}\left(r_{n}\right). \end{array}$$

Therefore,

$$\begin{array}{cc} \pi \left(\alpha \right)-\pi \left(\alpha^{\prime }\right) & =\underset{n\to \infty }{lim inf}\frac{1}{n}H_{\alpha }\left(r_{n}\right)-\lim_{k\to \infty }\frac{1}{n_{k}}H_{\alpha^{\prime }}\left(r_{n_{k}}\right)\\ \; & \leq \underset{k\to \infty }{lim inf}\frac{1}{n_{k}}\left(H_{\alpha }\left(r_{n_{k}}\right)-H_{\alpha^{\prime }}\left(r_{n_{k}}\right)\right). \end{array}$$

Note that $H_{\alpha }$ is a continuous function of the order α for α>1. We use the mean value theorem to claim that there is a value $\gamma_{k}\in (\alpha ,\alpha^{\prime }$ such that $H_{\alpha^{\prime }}\left(r_{n_{k}}\right)-H_{\alpha }\left(r_{n_{k}}\right)=\left(\alpha^{\prime }-\alpha \right)\frac{d}{d\alpha }H_{\gamma_{k}}\left(r_{n_{k}}\right).$ Next, for any probability vector *P*,

$$-\frac{dH_{\alpha }\left(P\right)}{d\alpha }=\frac{1}{{\left(1-\alpha \right)}^{2}}D\left(Z∥P\right)\leq \frac{\log|\mathrm{supp}(P)|}{{\left(1-\alpha \right)}^{2}},$$

where $Z_{i}=\frac{P_{i}^{\alpha }}{\sum_{j}P_{j}^{\alpha }}.$ Taking these remarks together, we obtain

$$\begin{array}{cc} \pi \left(\alpha \right)-\pi \left(\alpha^{\prime }\right) & \leq \underset{k\to \infty }{lim inf}\frac{1}{n_{k}}\left(\alpha -\alpha^{\prime }\right)H_{\gamma_{k}}^{\prime }\left(r_{n_{k}}\right)\\ \; & \leq \underset{k\to \infty }{lim inf}\frac{1}{n_{k}}\left(\alpha^{\prime }-\alpha \right)\frac{n_{k}}{{\left(\gamma_{k}-1\right)}^{2}}\\ \; & =\frac{\alpha^{\prime }-\alpha }{{\left(\alpha -1\right)}^{2}}, \end{array}$$

Therefore, π(α) is continuous on (1,∞). □

Now, let α∈(1,∞) and assume R>1−π(α). Choose $\alpha^{\prime }>\alpha$ such that $\alpha^{\prime }$ is rational and $R>1-\pi (\alpha^{\prime })$. This is possible from the continuity of π. Therefore,

$$\begin{array}{c} 1\leq \underset{n}{lim sup}Q_{n}\left(\alpha \right)\leq \underset{n}{lim sup}Q_{n}\left(\alpha^{\prime }\right)=1, \end{array}$$

which proves that (A8) and Theorem 3 hold for all α∈[1,∞).

It remains to address the case α=∞. We obtain the following upper bound, whose proof follows closely an argument in Appendix E of [3].

**Lemma A4.** 
*Let ϵ>0. We have*

$$\begin{array}{c} E_{C}{∥2^{n}T_{r}f_{C}∥}_{\infty }\leq 1+\epsilon +2^{2n-H_{\infty }\left(r\right)}e^{-\frac{3\epsilon^{2}}{2(3+\epsilon )}2^{-[n\left(1-R\right)-H_{\infty }\left(r\right)]}}. \end{array}$$

**Proof.** Let ϵ>0, then,

(A9) $$\begin{array}{cc} E_{C}{∥2^{n}T_{r}f_{C}∥}_{\infty }\; & =E_{C}\left[∥2^{n}T_{r}f_{C}{∥}_{\infty }1_{∥2^{n}T_{r}f_{C}{∥}_{\infty }\geq 1+\epsilon \}}\right]\\ \; & \;\;+E_{C}\left[∥2^{n}T_{r}f_{C}{∥}_{\infty }1_{∥2^{n}T_{r}f_{C}{∥}_{\infty }<1+\epsilon \}}\right]\\ \; & \leq E_{C}\left[∥2^{n}T_{r}f_{C}{∥}_{\infty }1_{\left\{∥2^{n}T_{r}f_{C}{∥}_{\infty }\geq 1+\epsilon \right\}}\right]+1+\epsilon \\ \; & \leq E_{C}\left[∥2^{n}{r∥}_{\infty }1_{\left\{∥2^{n}T_{r}f_{C}{∥}_{\infty }\geq 1+\epsilon \right\}}\right]+1+\epsilon \\ \; & =∥2^{n}{r∥}_{\infty }{\mathrm{Pr}}_{C}\left(\max_{y\in H_{n}}2^{n}T_{r}f_{C}\left(y\right)\geq 1+\epsilon \right)+1+\epsilon \\ \; & \leq ∥2^{n}{r∥}_{\infty }2^{n}\max_{y\in H_{n}}{\mathrm{Pr}}_{C}\left(2^{n}T_{r}f_{C}\left(y\right)\geq 1+\epsilon \right)+1+\epsilon . \end{array}$$

For any $y\in H_{n}$,

(A10) $$\begin{array}{cc} {\mathrm{Pr}}_{C}\left(2^{n}T_{r}f_{C}\left(y\right)\geq 1+\epsilon \right) & ={\mathrm{Pr}}_{C}\left(\frac{2^{n}}{M}\sum_{z\in C}r\left(y-z\right)\geq 1+\epsilon \right)\\ \; & ={\mathrm{Pr}}_{c_{i}∼U_{n},\;\mathrm{iid}\;}\left(\frac{2^{n}}{M}\sum_{i=1}^{M}r\left(y-c_{i}\right)\geq 1+\epsilon \right)\\ \; & =\mathrm{Pr}\left(\frac{2^{n}}{M}\sum_{i=1}^{M}r\left(y-c_{i}\right)\geq 1+\epsilon \right)\\ \; & =\mathrm{Pr}\left(\sum_{i=1}^{M}\left(2^{n}r\left(y-c_{i}\right)-1\right)\geq M\epsilon \right) \end{array}$$

To bound the last line from above, we use Bernstein’s inequality: For independent, zero-mean random variables $X_{i},i=1,\ldots ,N$ such that $|X_{i}|\leq a$ for all *i*,

$$P\left(\sum_{i}X_{i}\geq t\right)\leq \exp\left(-\frac{t^{2}/2}{\sum_{i=1}^{n}EX_{i}^{2}+\frac{1}{3}at}\right).$$

Note that for a random uniform vector $c_{i}$, the expectation $E\left[r\left(y-c_{i}\right)\right]=2^{-n}$ since r(·) satisfies $\sum_{x\in H_{n}}r\left(x\right)=1,$ so this inequality applies for (A10). We obtain

$$\begin{array}{cc} {\mathrm{Pr}}_{C}\left(2^{n}T_{r}f_{C}\left(y\right)\geq 1+\epsilon \right) & \leq \exp\left(-\frac{\frac{1}{2}M^{2}\epsilon^{2}}{\sum_{i=1}^{M}\mathrm{Var}\left(2^{n}r\left(y-\cdot \right)\right)+\frac{1}{3}{∥2^{n}r∥}_{\infty }M\epsilon }\right)\\ \; & \leq \exp\left(-\frac{\frac{1}{2}M^{2}\epsilon^{2}}{\sum_{i=1}^{M}∥2^{n}{r∥}_{2}^{2}+\frac{1}{3}{∥2^{n}r∥}_{\infty }M\epsilon }\right)\\ \; & \leq \exp\left(-\frac{\frac{1}{2}M^{2}\epsilon^{2}}{M∥2^{n}{r∥}_{1}∥2^{n}{r∥}_{\infty }+\frac{1}{3}{∥2^{n}r∥}_{\infty }M\epsilon }\right)\\ \; & =\exp\left(-\frac{3\epsilon^{2}}{2(3+\epsilon )}2^{-n\left(1-R\right)-H_{\infty }\left(r\right)}\right). \end{array}$$

where on the last line, we use the equalities $∥2^{n}{r∥}_{1}=1$ and $∥2^{n}{r∥}_{\infty }=2^{D_{\infty }\left(r∥U_{n}\right)}.$ The proof is concluded by substituting this inequality into (A9). □

Now, let us consider a sequence of (ensembles of) random codes of increasing length *n* and rate $R_{n}\to R$. Recalling the definition of π(·) in (15), for n→∞, we obtain

(A11) $$\begin{array}{c} \underset{n}{lim sup}E_{C_{n}}{∥2^{n}T_{r_{n}}f_{C_{n}}∥}_{\infty }\leq 1+\epsilon \end{array}$$

once R>1−π(∞). Since ϵ is arbitrarily small, the left-hand side of (A11) approaches one, and together with (14) this completes the proof of Theorem 3.

## Appendix C. Samorodnitsky’s Inequalities and Their Implications

Samorodnitsky [8,10] recently proved certain powerful inequalities for α-norms of noisy functions, which permit us to estimate the proximity to uniformity upon action of the Bernoulli noise kernels. We state some of them in this appendix after introducing a few more elements of notation. These results are used in Theorem 7 and in Appendix D, where we prove Lemma 2.

In this proof, we write [n] for {1,…,n}. For a subset Γ⊂[n], write ${x|}_{\Gamma }$ to denote the coordinate projection of a vector $x\in H_{n}$ on Γ. If the subset Γ is formed by random choice with Pr(i∈Γ)=λ independently for all i∈[n], we write Γ∼λ. For a function *f* on $H_{n}$, let

(A12) $$E\left(f|\Gamma \right)\left(x\right)=\frac{1}{2^{n-|\Gamma |}}\sum_{{y:y|}_{\Gamma }{=x|}_{\Gamma }}f\left(y\right).$$

Observe that $E\left(f|\Gamma \right)=f*f_{H_{[n]\setminus \Gamma }},$ where $H_{S}=\left\{x\in H_{n}:x{|}_{[n]\setminus S}=0\right\}$. Therefore, E(f|Γ)(x) is the noisy function of *f* with respect to the pmf given by the indicator function of the subcube $H_{[n]\setminus \Gamma }$.

The *entropy* of a function $f:H_{n}\to R$ is defined as

(A13) $$\mathrm{Ent}\left[f\right]={∥f\log f∥}_{1}-{∥f∥}_{1}{\log(∥f∥}_{1})=∥f\log\frac{f}{{∥f∥}_{1}}{∥}_{1}.$$

This quantity can be thought of as the KL divergence between the distribution induced by *f* on $H_{n}$ and the uniform distribution:

(A14) $$\mathrm{Ent}\left[f\right]={∥f∥}_{1}D\left(\frac{f}{\sum f}∥U_{n}\right).$$

If *f* itself is a pmf, then $D\left(f∥U_{n}\right)=2^{n}\mathrm{Ent}\left(f\right)=\mathrm{Ent}\left(2^{n}f\right).$

**Theorem A2** ([8], Corollary 9). *Let f be a non-negative function on $H_{n}$. then,*

$$\begin{array}{c} \mathrm{Ent}\left[T_{\delta }f\right]\leq E_{\Gamma ∼\lambda }\mathrm{Ent}\left[E\left(f|\Gamma \right)\right]. \end{array}$$

*where $\lambda ={\left(1-2\delta \right)}^{2}.$*

**Theorem A3** ([10], Theorem 1.1). *Let f be a non-negative function on $H_{n}$ and α≥2 be an integer. Then,*

(A15) $$\log∥T_{\delta }{f∥}_{\alpha }\leq E_{\Gamma ∼\lambda }\log{∥E\left(f|\Gamma \right)∥}_{\alpha }.$$

*where $\lambda =\lambda \left(\alpha ,\delta \right)=1+\frac{1}{\alpha -1}\log\left(\delta^{\alpha }+{\left(1-\delta \right)}^{\alpha }\right)=1-h_{\alpha }\left(\delta \right)$. Furthermore,*

(A16) $$\log∥T_{\delta }{f∥}_{\infty }\leq E_{\Gamma ∼\lambda }\log{∥E\left(f|\Gamma \right)∥}_{\infty }.$$

*where $\lambda =\lambda \left(\infty ,\delta \right)=1+\log\left(1-\delta \right)=1-h_{\infty }\left(\delta \right)$*

To interpret the inequalities (A15) and (A16), we note that their left-hand side measures the smoothness of the noisy version of *f* with respect to the noise $\beta_{\delta }$. At the same time, the right-hand side is the average smoothness of the noisy versions of *f* with respect to the sub-cube pmf’s.

Hązła et al. [14] used Theorem A3 to great effect, showing that if a code corrects erasures up to a certain noise level in a BEC, then, with high probability, it corrects errors on a BSC channel up to a certain noise level.

**Theorem A4** ([14], Corollary 3.4). *Let ${\left(C_{n}\right)}_{n}$ be a sequence of codes whose rate approaches R. Assume that for some λ∈(0,1−R], $P_{B}\left(\mathrm{BEC}\left(\lambda \right),C_{n}\right)=o\left(\frac{1}{n}\right)$. Then, ${\left(C_{n}\right)}_{n}$ decodes errors on a BSC(δ) for any δ that satisfies $2\sqrt{\delta (1-\delta )}<2^{\lambda }-1.$*

This theorem implies the following corollary:

**Corollary A1** ([14]). *Let ${\left(C_{n}\right)}_{n}$ be a sequence of codes with rate $R_{n}↑R$ that recover transmitted messages with high probability on a BEC(1−R) (i.e., ${\left(C_{n}\right)}_{n}$ is a capacity-achieving sequence for BEC(1−R)). Furthermore, assume that $d\left(C_{n}\right)=\omega \left(\log n\right)$. If $2\sqrt{\delta (1-\delta )}<2^{1-R}-1$, then with high probability, the codes $C_{n}$ correct errors when used on a BSC(δ) channel.*

The authors of [14] then used this result to show that the RM codes of a constant rate correct a non-vanishing proportion of errors on the BSC.

## Appendix D. Proof of Lemma 2

We present the proof as a sequence of lemmas.

Let Γ⊂{1,…,n} be a subset of coordinates and for $z\in {\left(0,1\right\}}^{n}$ let $C\left(\Gamma ,z\right):=\{c\in C:c{|}_{\Gamma }=z{|}_{\Gamma }\}$ be the set of codewords that fit *z* in the positions of Γ. In particular, $C^{\Gamma^{c}}{=C\left(\Gamma ,0\right)|}_{\Gamma^{c}}$ is the shortened code C, i.e., the subcode with zeros in the positions of Γ, projected on $\Gamma^{c}.$ Let $F^{\left(C\right)}\left(\Gamma ,z\right):=\left|C\left(\Gamma ,z\right)\right|$.

Let us obtain expressions for the norms and the entropy of $F^{\left(C\right)}\left(\Gamma ,z\right).$

**Lemma A5.** 
*Let C be a linear code and let Γ⊂{1,…,n}. Then,*

$$\begin{array}{c} ∥F^{\left(C\right)}{\left(\Gamma ,\cdot \right)∥}_{\alpha }={\left[\frac{\left|C\right|}{2^{\left|\Gamma \right|}}F^{\left(C\right)}{\left(\Gamma ,0\right)}^{\alpha -1}\right]}^{1/\alpha }. \end{array}$$

**Proof.** From the linearity of the code,

$$F^{\left(C\right)}\left(\Gamma ,z\right)=\left\{\begin{array}{cc} F^{\left(C\right)}\left(\Gamma ,0\right) & {\mathrm{if}z|}_{\Gamma }\;\mathrm{is a valid non}-\mathrm{erasure pattern}\\ 0 & \mathrm{otherwise}. \end{array}\right..$$

Furthermore, the number of distinct $z\in H_{n}$ for which C(Γ,z) is nonempty equals $2^{n-|\Gamma |}\left|C/C\left(\Gamma ,0\right)\right|.$ Hence,

$$\begin{array}{cc} ∥F^{\left(C\right)}{\left(\Gamma ,\cdot \right)∥}_{\alpha } & ={\left[\frac{1}{2^{n}}\sum_{x\in H_{n}}F^{\left(C\right)}{\left(\Gamma ,x{|}_{\Gamma }\right)}^{\alpha }\right]}^{1/\alpha }\\ \; & ={\left[\frac{1}{2^{n}}\frac{2^{n}\left|C\right|}{2^{\left|\Gamma \right|}}\frac{1}{F^{\left(C\right)}\left(\Gamma ,0\right)}F^{\left(C\right)}{\left(\Gamma ,0\right)}^{\alpha }\right]}^{1/\alpha }\\ \; & ={\left[\frac{\left|C\right|}{2^{\left|\Gamma \right|}}F^{\left(C\right)}{\left(\Gamma ,0\right)}^{\alpha -1}\right]}^{1/\alpha }. \end{array}$$

□

**Lemma A6.** 
*Let E(f|Γ) be defined as in (A12). Then,*

$$\begin{array}{cc} ∥E\left(2^{n}f_{C}|\Gamma \right){∥}_{\alpha }\; & ={\left[\frac{2^{\left|\Gamma \right|}}{\left|C\right|}F_{T}^{\left(C\right)}\left(\Gamma ,0\right)\right]}^{(\alpha -1)/\alpha }. \end{array}$$

**Proof.** Using $f=2^{n}f_{C}$ in (A12), we obtain

$$\begin{array}{cc} E\left(2^{n}f_{C}|\Gamma \right)\left(x\right) & =\frac{2^{n}}{2^{n-|\Gamma |}\left|C\right|}\sum_{{y\in C:y|}_{\Gamma }{=x|}_{\Gamma }}1=\frac{2^{\left|\Gamma \right|}}{\left|C\right|}F^{\left(C\right)}\left(\Gamma ,x{|}_{\Gamma }\right), \end{array}$$

and, thus, from Lemma A5,

$$\begin{array}{cc} ∥E\left(2^{n}f_{C}|\Gamma \right){∥}_{\alpha } & =\frac{2^{\left|\Gamma \right|}}{\left|C\right|}{∥F^{\left(C\right)}\left(\Gamma ,\cdot \right)∥}_{\alpha }\\ \; & =\frac{2^{\left|\Gamma \right|}}{\left|C\right|}{\left[\frac{\left|C\right|}{2^{\left|\Gamma \right|}}F^{\left(C\right)}{\left(\Gamma ,0\right)}^{\alpha -1}\right]}^{1/\alpha }\\ \; & ={\left[\frac{2^{\left|\Gamma \right|}}{\left|C\right|}F_{T}^{\left(C\right)}\left(\Gamma ,0\right)\right]}^{(\alpha -1)/\alpha }. \end{array}$$

□

**Lemma A7.** 
*Let C be a linear code. For $X=X_{C^{⊥}},Y=Y_{\left(X,\mathrm{BEC}(\lambda )\right)}$,*

(A17)
$$\begin{array}{c} H\left(X|Y\right)=E_{\Gamma ∼\lambda }\left[\log\left(\frac{2^{\left|\Gamma \right|}}{\left|C\right|}F^{C}\left(\Gamma ,0\right)\right)\right]. \end{array}$$

**Proof.** Start with taking $X=X_{C}$ and $Y=Y_{\left(\mathrm{BEC}\left(\lambda \right),X_{C}\right)}$; then,

$$\begin{array}{c} H\left(X|Y=y\right)=\log[F^{\left(C\right)}\left(y\right)]. \end{array}$$

Therefore,

$$\begin{array}{cc} H(X|Y) & =E_{Y}\left[\log\left(F^{\left(C\right)}\left(Y\right)\right)\right]\\ \; & =E_{\Gamma }E_{Z|\Gamma }\left[\log\left(F^{\left(C\right)}\left(\Gamma ,Z\right)\right)|\Gamma \right]\\ \; & =E_{\Gamma ∼1-\lambda }\left[\log F^{\left(C\right)}\left(\Gamma ,0\right)\right]. \end{array}$$

By a standard identity about dual matroids ([64], p. 72),

$$\dim\left(C^{\Gamma^{c}}\right)=\dim\left(C\right)-\left|\Gamma \right|+\dim\left({\left(C^{⊥}\right)}^{\Gamma }\right),$$

or

$$F^{\left(C\right)}\left(\Gamma ,0\right)=\frac{\left|C\right|}{2^{\left|\Gamma \right|}}F^{C^{⊥}}\left(\Gamma^{c},0\right),$$

and, thus, we continue as follows:

$$\begin{array}{cc} H(X|Y) & =E_{\Gamma ∼1-\lambda }\left[\log\left(\frac{\left|C\right|}{2^{\left|\Gamma \right|}}F^{C^{⊥}}\left(\Gamma^{c},0\right)\right)\right]\\ \; & =E_{\Gamma^{c}∼\lambda }\left[\log\left(\frac{2^{|\Gamma^{c}|}}{|C^{⊥}|}F^{C^{⊥}}\left(\Gamma^{c},0\right)\right)\right]\\ \; & =E_{\Gamma ∼\lambda }\left[\log\left(\frac{2^{\left|\Gamma \right|}}{|C^{⊥}|}F^{C^{⊥}}\left(\Gamma ,0\right)\right)\right]. \end{array}$$

Switching to the dual code and taking $X=X_{C^{⊥}}$ and $Y=Y_{\left(\mathrm{BEC}\left(\lambda \right),X_{C^{⊥}}\right)}$ now yields (A17). □

**Lemma A8.** 

$$\begin{array}{c} \frac{\alpha }{\alpha -1}E_{\Gamma ∼\lambda }\log{∥E\left(2^{n}f_{C}|\Gamma \right)∥}_{\alpha }=E_{\Gamma ∼\lambda }\mathrm{Ent}\left[E\left(2^{n}f_{C}|\Gamma \right)\right]=H\left(X_{C^{⊥}}|Y_{\left(\mathrm{BEC}\left(\lambda \right),X_{C^{⊥}}\right)}\right). \end{array}$$

**Proof.** From Lemmas A6 and A7,

$$\begin{array}{cc} \frac{\alpha }{\alpha -1}E_{\Gamma ∼\lambda }\log{∥E\left(2^{n}f_{C}|\Gamma \right)∥}_{\alpha }\; & =E_{\Gamma ∼\lambda }\left[\log\left(\frac{2^{\left|\Gamma \right|}}{\left|C\right|}F^{C}\left(\Gamma ,0\right)\right)\right]\\ \; & =H(X_{C^{⊥}}|Y_{\left(\mathrm{BEC}\left(\lambda \right),X_{C^{⊥}}\right)}), \end{array}$$

which establishes the equality between the first and the third quantities. Since the second quantity is a limiting case of the first quantity and the value of the first quantity is independent of α, we have equality between the first and the second quantities. □

Now, Lemma 2 follows by combining Lemma A8 with Theorems A2 and A3.
